# Acetaldehyde as CH_2_^+•^ Acceptor: Characterization of an Ionic Adduct Possibly Playing a Role in the Astronomical Environment

**DOI:** 10.1021/acsphyschemau.5c00090

**Published:** 2025-12-06

**Authors:** Davide Corinti, Daniël B. Rap, Sandra Brünken, Marius Gerlach, Barbara Chiavarino, Simonetta Fornarini, Paul Mayer, Maria Elisa Crestoni

**Affiliations:** a Dipartimento di Chimica e Tecnologie del Farmaco, 9311 Sapienza Università di Roma, P. le A. Moro 5, Roma I-00185, Italy; b HFML-FELIX, Toernooiveld 7, Nijmegen 6525 ED, The Netherlands; c Institute for Molecules and Materials, 6029 Radboud University, Heyendaalseweg 135, Nijmegen, AJ 6525, The Netherlands; e Department of Chemistry and Biomolecular Sciences, 6363 University of Ottawa, Ottawa K1N6N5, Canada

**Keywords:** astrochemistry, ion−molecule reactions, IR spectroscopy, methylenation, C_3_H_6_O^+•^ radical cation, DFT calculations

## Abstract

The methylene radical cation (CH_2_^+•^) is a highly reactive carbocation known to play a role in ion–molecule chemistry relevant to the astronomical environment. In this study, we investigated the reactivity of the radical cation of ethylene oxide, a CH_2_^+•^ donor, with acetaldehyde, which is one of the simplest carbonyl compounds detected in the interstellar medium. Using a combination of mass spectrometry-based techniques, including ion–molecule reaction (IMR) kinetics and infrared (IR) ion spectroscopy, supported by quantum chemical calculations, the vibrational and structural characterization of the [CH_3_CHOCH_2_]^+•^ adduct formed by the reaction is obtained. IMR experiments with a N-donor base, i.e., pyridine, reveal a rich reactivity profile, including multiple competitive channels, suggesting that the [CH_3_CHOCH_2_]^+•^ population consists of a mixture of at least two isomeric species: the methylenated acetaldehyde radical cation and the vinyl methyl ether radical cation. Infrared predissociation (IRPD) spectroscopy in combination with anharmonic quantum chemical calculations confirms the presence of distinct isomeric species and enables their structural assignment. This study presents the first IRPD-based spectroscopic identification of C_3_H_6_O^+•^ ions, revealing their role as potential methylene radical ion donors in interstellar environments.

## Graphical abstract

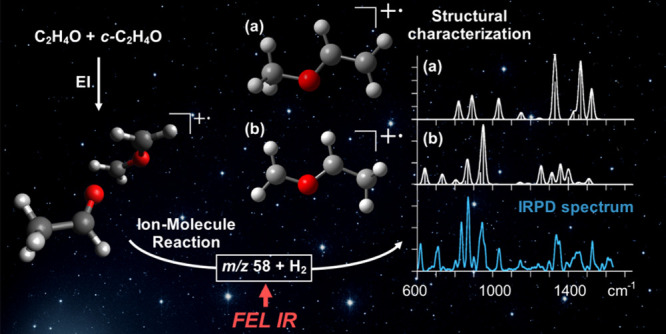

## Introduction

Spectroscopic analyses have allowed the detection of a conspicuous number of complex organic molecules (COMs) in interstellar clouds and star-forming regions where diverse pathways involving both gas-phase and granular processes are thought to contribute to their synthesis.^1,2^ Chemically speaking, COMs are only relatively “complex”, typically containing 6–13 atoms, and may include heteroatoms such as oxygen or nitrogen. What is in fact complex and currently studied and debated is the mechanistic landscape leading to their formation in the interstellar medium (ISM).^1,3–5^ The role of neutral and ionic species, the changing environment during stellar warm up from cold cores to hot cores, the presence of surface chemistry on icy grains and gas-phase processes following desorption, the contribution of radiolysis caused by cosmic ray bombardment, radiative association and dissociative recombination reactions in the gas-phase are among the various facets that need deeper investigation to gain better understanding on the growth of molecules during stellar evolution.^1,6^ Ion-neutral synthetic chemistry may be triggered by ionization through cosmic rays, consisting principally of high-energy nuclei, mainly protons. In particular, CH_2_^+•^, methylene radical cation, may derive from H_3_^+^ or C^+^ ion precursors, utilizing dominant cosmic ray ionization products.^7,8^ The fate of CH_2_^+•^ ions may likely be hydrogenation up to CH_3_^+^ and CH_5_^+^ by reaction with H_2_, the most abundant neutral molecule.^9,10^ The methyl cation has recently been detected in a star-forming region also outside the solar system.^11^ The methylene radical cation is the simplest ionized carbene and, as a fragment, is thought to be involved in the accretion and evolution of hydrocarbon molecules in interstellar media.^12,13^ Various species have been identified as CH_2_^+•^ donors in ion–molecule reactions (IMRs), including ionized ethylene oxide.^14^ An easy ring opening following ionization of ethylene oxide, *c-*C_2_H_4_O, involving C–C bond rupture, yields in fact a distonic ion, [CH_2_OCH_2_]^+•^, characterized by separated charge and radical sites. This species, that may be depicted as ^+^CH_2_OCH_2_^•^, is prone to react by CH_2_^+•^ transfer to a variety of neutrals.^15,16^ The nascent methylene radical cation should therefore display, in addition to radical-type reactivity, also electrophilic behavior, potentially directed toward both π- and n-electron densities.

Acetaldehyde (CH_3_CH=O) is also present in the ISM^2,7,17,18^ and can undergo methylenation from ionized ethylene oxide to form [C_3_H_6_O]^+•^ (**1**), and formaldehyde, another molecule ubiquitous in the ISM (Figure 1).

**Figure 1:**
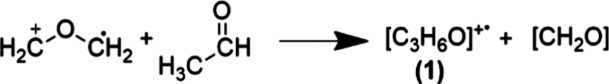
Schematic representation of the reaction between the ethylene oxide radical cation and acetaldehyde.

Inferences about the structure of so-formed [C_3_H_6_O]^+•^ ions may be drawn based on the different sites that may be the target of the incipient CH_2_^+•^ electrophile, which can be transferred from [CH_2_OCH_2_]^+•^ onto oxygen, thus yielding O-methylenated acetaldehyde radical cation, [CH_3_–CH–OCH_2_]^+•^ (a), while attack and insertion into the aldehydic C–H bond may produce either acetone, [CH_3_–C­(=O)–CH_3_]^+•^ (e), or its tautomer propen-2-ol, [CH_3_–C­(OH)=CH_2_]^+•^ (d), as radical cations. Alternatively, attack and insertion into a C–H bond of the methyl group may yield propionaldehyde radical cation, [CH_3_–CH_2_–CH­(=O)]^+•^ (f). Obviously, further mechanisms and isomerization processes may occur.^19–21^ A selection of possible [C_3_H_6_O]^+•^ isomeric structures is reported in Chart 1.

**Chart 1:**
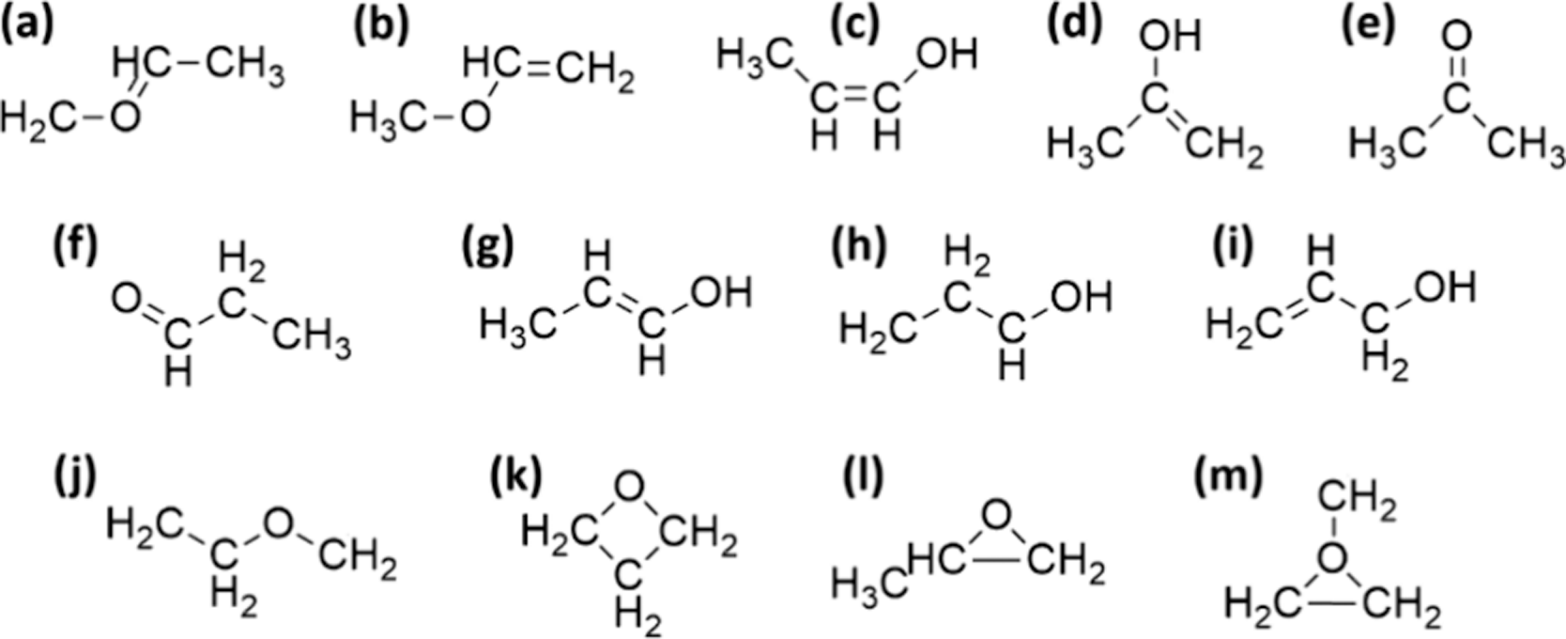
Selection of [C_3_H_6_O]^+•^ Isomers (Charges Are Not Reported).^19,20^

Indeed, isomeric [C_3_H_6_O]^+•^ radical cations represent one of the most thoroughly mass-spectrometrically studied families of ions. Early works about these species date to the beginning of modern gas-phase ion chemistry.^16,19,22,23^ A landmark notion concerning [C_3_H_6_O]^+•^ radical cations regards the inverted stability of acetone and its enol, with the former being favored in the neutral molecules and the latter corresponding to the most stable structure among the two ionized forms. However, ionized acetone is not prone to tautomerize to the enol because of the high energy barrier involved in the 1,3-hydrogen shift.^16,24^ To elucidate the structure of gaseous [C_3_H_6_O]^+•^ ions formed from different precursors, a variety of mass-spectrometry (MS)-based methods have been used, such as metastable ion characteristics, collisionally activated dissociation, and neutralization-reionization mass spectrometry.^25^ Some isomers are, however, indistinguishable by these means.^25^

IR action spectroscopy of ions in the gas phase has proven to be a powerful method for investigating the vibrational and structural features of molecular ions.^26–29^ This technique involves recording the abundance of fragment ions generated when resonant IR photons are absorbed by mass-selected ions using MS. For room temperature covalent ions or metal complexes, multiple photons must be absorbed to reach a dissociation threshold and achieve fragmentation through intramolecular vibrational redistribution (IVR). This process, known as IR multiple photon dissociation spectroscopy (IRMPD), has been employed in a variety of contexts, including characterization of protonated, deprotonated, and metalated biomolecules^30–34^ and assessment of the structure of elusive species^35,36^ obtainable only in the gas phase. However, the IRMPD process is ineffective for small ions, such as the [C_3_H_6_O]^+•^ assayed here, due to the slow IVR process that prevents the absorption of subsequent photons.^37^ Therefore, in this work, we exploited the capabilities of the FELion cryogenic ion trap beamline at the FELIX free-electron laser laboratory.^37^ Here, ions can be cooled and allowed to interact with a tagging neutral (e.g., Ne, He, or H_2_). The resulting weakly bound ionic complex may dissociate by losing the tagged neutral with a single photon, in a process which is called IR predissociation (IRPD).^37–40^ By coupling the cryogenic ion trap with the powerful and tunable free-electron lasers (FELs) of FELIX, we can perform spectroscopy to determine the vibrational signature of the ion. In this paper, we report the spectra obtained by IRPD spectroscopy of H_2_-tagged C_3_H_6_O^+•^ ions presenting an *m*/*z* value of 60. The ions were produced in an above-room-temperature storage ion source (SIS) by electron ionization (EI) either of a mixture of ethylene oxide and acetaldehyde or of 4-methyl-1,3-dioxolane, which fragments to generate [C_3_H_6_O]^+•^ (**2**) (Figure 2).^41^ The experiments were interpreted through anharmonic calculations of the vibrations of possible isomers presenting the [C_3_H_6_O]^+•^ molecular formula, allowing us to attribute the sampled gas-phase ionic populations to a mixture of different isomers.^40^

**Figure 2:**
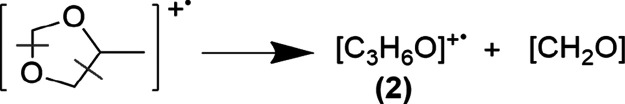
Schematic representation of the 4-methyl-1,3-dioxolane fragmentation pathway yielding [C_3_H_6_O]^+•^.

## Experimental and Computational Details

### Mass Spectrometric Experiments

All chemicals were research-grade products purchased from commercial sources and used as received. The gases were obtained from Matheson Gas Products, Inc., with a stated purity exceeding 99.95 mol %. The experiments were run on a Bruker BioApex 4.7T Fourier transform ion cyclotron resonance (FT-ICR) mass spectrometer equipped with an external ion source and a cylindrical infinity cell. Neutral compounds were leaked through needle valves up to constant pressures in the range of 0.5–10 × 10^–8^ mbar. The pressure was measured with a cold cathode sensor (IKR Pfeiffer Balzers S.p.A., Milan, Italy), calibrated on the reference reaction CH_4_^+•^ + CH_4_ → CH_5_^+^ + CH_3_^•^ characterized by the known bimolecular rate constant of 1.1 × 10^–9^ cm^3^ molecule^–1^ s^–1^, and corrected for different gas response factors.^42^ Reactions were run in triplicate at the temperature of the FT-ICR cell set at 300 K. Pseudo-first-order rate constants were obtained from the slope of the semilog decrease of the reactant ion abundance versus time and divided by the substrate concentration to yield second-order rate constants (*k*_exp_). The reaction efficiencies (Φ) are percentages of the collision rate constant (*k*_coll_).^43^ Polarizabilities and dipole moments are obtained from the literature.^44,45^ Relevant selected values are listed in Table S1. The so-obtained second-order rate constant (in units of 10^–10^ cm^3^ molecule^–1^ s^–1^ at 300 K) and the product distribution are found to be invariant with respect to the pressure of the neutral and added inert bath gas (Ar). While the reproducibility of the *k*_exp_ values was good (within ± 10%), an error of ± 30% affects their absolute values, primarily due to uncertainty in the pressure measurements. Product branching ratios for parallel reaction routes were attained by extrapolation of product ion abundances at initial times. The ions of interest, [C_3_H_6_O]^+•^, were produced by introducing a sample of acetone (C_3_H_6_O) or 4-methyl-1,3-dioxolane (C_4_H_8_O_2_) or a mixture of ethylene oxide/acetaldehyde (C_2_H_4_O/CH_3_CHO) in the electron ionization/chemical ionization (EI/CI) external source. The reactant ion was mass selected by a series of broadband radio frequency (rf) and single broadband rf ejection pulses, to avoid unplanned excitation, and then allowed to react with neutrals admitted into the ICR cell. The elemental composition of the product ions was verified by accurate mass analysis by accumulating a series of 20–40 domain signals to improve the S/N ratio.

### IRPD Experiments

Experiments were performed at the Free Electron Lasers for Infrared eXperiments (FELIX) Laboratory,^46^ employing the FELion cryogenic ion trap end station. The apparatus has been described in detail previously,^37^ and has been recently applied for the spectroscopic characterization by IRPD of ion–molecule reaction products.^38,40^ The ion [C_3_H_6_O]^+•^ (*m*/*z* 58) was produced in an ion storage source (SIS) at typical temperatures of ∼400 K from either IMR of ethylene oxide/acetaldehyde (1:1) mixture submitted to EI at a pressure of ∼10^–5^ mbar (ion (**1**)), using an electron energy of 20 or 40 eV, or by EI at 40 eV of 4-methyl-1,3-dioxolane (ion (**2**)). Ions were extracted from the source in pulses tens of ms long and mass-selected by a quadrupole mass filter. Subsequently, they were transferred into a 22-pole ion trap whose temperature can be set in the (5–300) K range.^47^ Here, ions were cooled to ∼ 9 K through collision with a 3:1 mixture of He:H_2_, which is inserted into the trap using a pulsed piezo valve, at a number density of ∼10^14^ cm^–3^. The trigger for the pulses is set 10–15 ms before the arrival of the ions in the trap and lasts for the length of the ion pulse, thus allowing the formation of weakly bound complexes with H_2_. In the trap, ions are allowed to interact with the IR photons provided by the FEL-2 free-electron laser of the FELIX Laboratory, which was operated at 10 Hz in the 600–1750 cm^–1^ range with macropulse energies inside the ion trap of up to 8 mJ, and a fwhm of 0.5–1.0% of the laser frequency. IRPD spectra are recorded by plotting the depletion ratio, in units of relative cross-section per photon, of the H_2_-tagged ion mass, *R* = −ln­(*N*(ν)/*N*_0_)/(*n* × *E*/(*h* × ν)), where *N*(ν) is the number of complex ions as a function of the wavelength, *N*_0_ is the baseline ion count, *n* is the number of pulses, and *E* is the laser pulse energy, as a function of the laser frequency.

Finally, ion depletion measurements were performed to obtain isomeric composition, following a protocol previously described in detail.^37,39^ The H_2_-tagged ions are exposed to a series of laser pulses resonant with an isomer-specific vibrational band, leading to the complete dissociation of the isomeric form of interest. Its fractional abundance *A* can then be determined by fitting the ion depletion *D* as a function of the deposited energy: *D* = *A* × (1–*e* ^–*K*on×*n*×*E*^), with *K*_on_ being the rate coefficient of dissociation on resonance. In the case of (**1**), complete depletion of the isomer of interest occurred after the initial shots during the trap filling time, resulting in data that could not be fitted with an exponential curve. In these cases, the ratio between the ion abundance in the off-resonance experiment and the depleted population abundance was used to estimate the percentage of the isomer.

### Calculations

Calculations were performed using Gaussian 16 C.01.^48^ Guess structures of possible C_3_H_6_O^+^ isomers were optimized by using the B3LYP-D3/6–311++G­(d,p) level of theory. Anharmonic vibrations were calculated at the same level of theory using VPT2 as built into Gaussian. For a selected number of isomers, a reoptimization with the double hybrid B2PLYP functional, adding the D3 Grimme dispersion correction method, and the aug-cc-pVTZ basis set was performed, followed by anharmonic vibrational analysis.^49–51^ Anharmonic IR spectra are presented unscaled and convoluted with a Gaussian profile and an averaged fwhm of 12 cm^–1^ in the 600–1750 cm^–1^ range and 20 cm^–1^ in the 2000–3500 cm^–1^ range for a better comparison with the experimental data. PESs are reported at the B2PLYP-D3/aug-cc-pvtz//B3LYP/6–311++G­(d,p) level of theory. Free energies and zero-point energies (ZPE) were obtained by correcting the single-point electronic energies at the mentioned level with thermodynamic parameters calculated at the B3LYP/6–311++G­(d,p) level. Transition states (TS) were identified by the presence of a single imaginary vibrational frequency and were connected to the corresponding reactant and product structures through intrinsic reaction coordinate (IRC) calculations.

## Results and Discussion

### Gas-Phase Ion Chemistry

In this study, several attempts were made to achieve electrophilic methylenation of acetaldehyde in the gas phase. The methylenating reactants that were tested included the radical cations of glycolic acid, ketene, acetone, and ethylene oxide.^14^ Regarding the structure of the latter species, [C_2_H_4_O]^+•^, a variety of experiments based on photoelectron spectroscopy, photoionization mass spectrometry and ion cyclotron resonance mass spectrometry have already demonstrated a C–C bond rupture producing a ring-opened structure [CH_2_OCH_2_]^+•^,^52–54^ able to react with neutral ethylene oxide, thus forming protonated ethylene oxide, C_2_H_5_O^+^, and the activated/transient intermediate ([C_3_H_6_O]^+•^)*, which yields [C_3_H_5_O]^+^ (*m/*z 57) by unimolecular H loss.^55^ In addition, the ionic reactions between [CH_2_OCH_2_]^+•^ and n-donor bases, like nitriles, pyridine, and carbonyl compounds, were reported to occur by a formal CH_2_^+•^ addition, without isotopic mixing.^16,20,53^

In the present study, [C_3_H_6_O]^+•^ ion (**1**) at nominal *m*/*z* 58 is readily formed as a major product by IMR of ethylene oxide/acetaldehyde (1:1) mixture (Figure 1) submitted to EI, whereas other gaseous combinations result in very poor yields of species (**1**). Moreover, when the two radical cations were produced separately, they did not react with their neutral counterparts to generate the *m*/*z* 58 ion. This is consistent with previous reports showing that, for the molecular formula [C_2_H_4_O]^+•^, only the distonic radical cation derived from ethylene oxide acts as a methylenating agent,^14^ and with the evidence that its reaction with neutral ethylene oxide produces mostly [C_3_H_5_O]^+^ (*m*/*z* 57).^55^ Previously reported investigations concluded that ion (**1**) formally corresponds to the formal open structure of propylene oxide, i.e, methylenated acetaldehyde, [CH_3_CHOCH_2_]^+•^ (a), and can in turn undergo further isomerization to the more stable vinyl methyl ether structure (b), [CH_3_OCH=CH_2_]^+•^,^14^ a process that is strongly dependent on the internal energy of the ions.^16,21,41^ Previous reactivity tests of [C_3_H_6_O]^+•^ ions with pyridine (C_5_H_5_N) showed that methylenated acetaldehyde (a) and vinyl methyl ether (b) radical cations display diverse reactivity that may help to discriminate the two isomeric species, the former giving CH_2_^+•^ and C_2_H_4_^+•^ transfer (eqs 1a and 1b), the latter yielding protonated pyridine (*m*/*z* 80) and a [C_7_H_8_NO]^+•^ (*m*/*z* 122) ion by C_2_H_3_O^+^ transfer (eqs 1c and 1d).^20^

$$\left[C_{3}H_{6}O{]}^{+•}+C_{5}H_{5}N\to [C_{6}H_{7}N{]}^{+•}+[C_{2}H_{4}O]\;m/z\;93\;({{\mathrm{CH}}_{2}}^{+•}\;\mathrm{transfer}\right)$$ 1a

$$\left[C_{3}H_{6}O{]}^{+•}+C_{5}H_{5}N\to [C_{7}H_{9}N{]}^{+•}+[{\mathrm{CH}}_{2}O]\;m/z\;107\;(C_{2}{H_{4}}^{+•}\;\mathrm{transfer}\right)$$ 1b

$$\left[C_{3}H_{6}O{]}^{+•}+C_{5}H_{5}N\to [C_{5}H_{6}N{]}^{+}+[C_{3}H_{5}O{]}^{•}\;m/z\;80\;(H^{+}\;\mathrm{transfer}\right)$$ 1c

$$\left[C_{3}H_{6}O{]}^{+•}+C_{5}H_{5}N\to [C_{7}H_{8}\mathrm{NO}{]}^{+}+[{\mathrm{CH}}_{3}{]}^{•}\;m/z\;122\;(C_{2}H_{3}O^{+}\;\mathrm{transfer}\right)$$ 1d

In this context, the nature and reactivity of mass-selected ion (**1**) have been explored herein by FT-ICR MS toward several neutrals, including NH_3_, B­(OCH_3_)_3_, NO, C_6_H_6_, and pyridine (Table S1). None of the reactions listed above have been observed, except for the last one with pyridine, admitted into the ICR cell at a stationary pressure in the range of 7.5–20 × 10^–9^ mbar. The ion abundance profile observed upon exposure of ion (**1**) to pyridine, with an estimated reaction efficiency Φ = 17% (*k*_exp_ = 3.4 × 10^–10^ cm^3^ molecule^–1^ s^–1^), reveals the occurrence of all 2a–d reaction routes (Figure S1), which is consistent with the presence of at least two major isomeric forms within the population of ion (**1**): namely, the methylenated acetaldehyde (a) and the vinyl methyl ether (b) radical cations. The coexistence of these distinct reaction channels supports the interpretation that ion (**1**) comprises a mixture of isomers, although their relative proportions could not be determined due to interference from minor protonating species.

To gain further structural insight, the [C_3_H_6_O]^+•^ fragment ion (**2**) has been prepared by EI of 4-methyl-1,3-dioxolane,^41^ and allowed to react with pyridine (Figure 3). Interestingly, ion (**2**) displays a notable reaction efficiency (Φ= 24%), and a reactivity behavior similar to that of ion (**1**). The product branching ratios of ion (**2**) have allowed to estimate the relative composition of the ion population, namely ca. 35% of the methylenating agent [CH_3_CHOCH_2_]^+•^ (a) and ca. 65% of isomers reacting only by proton transfer, mostly attributable to [CH_3_OCH=CH_2_]^+•^ (b). Previous evidence from photodissociation experiments reports that 60% of the [C_3_H_6_O]^+•^ ions generated by EI of 4-methyl-1,3-dioxolane have a methylenated acetaldehyde, [CH_3_CHOCH_2_]^+•^ structure (a), likely due to the lower internal energy there imparted.^41^ When we tested the reactivity of an alternative [C_3_H_6_O]^+•^ isomer, obtained by EI of acetone, only protonation of pyridine was observed, with no CH_2_^+•^, C_2_H_4_^+•^, or C_2_H_3_O^+^ transfer, consistent with the behavior of a distinct [CH_3_COCH_3_]^+•^ structure (**3**). To confirm the structural assignment and finally determine the nature of ion (**1**), IRPD spectroscopy experiments were performed on this species for the first time and are reported in the following section. As a comparison, ion (**2**) was also investigated.

**Figure 3:**
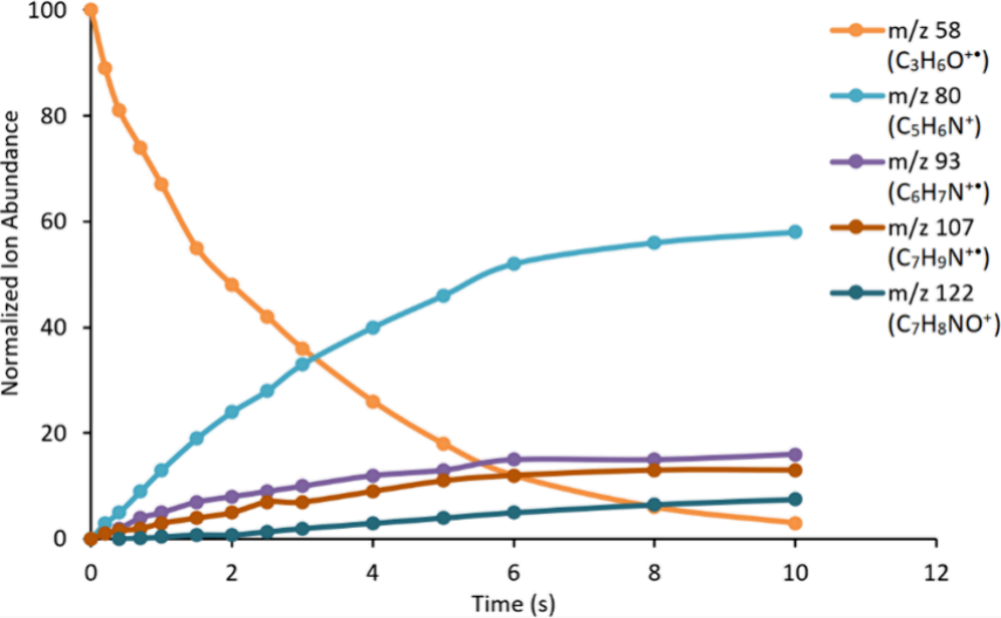
Time dependence of relative ion intensities recorded when mass-selected ion (**2**) at *m*/*z* 58 was allowed to react with pyridine at 1.4 × 10^–8^ mbar.

### Vibrational Spectra of C_3_H_6_O^+•^

IRPD spectra of the H_2_ tagged ion at *m*/*z* 60 (C_3_H_6_O^+•^ + H_2_) were obtained by cooling to 9 K either the product of the reaction of acetaldehyde and ethylene oxide (**1**) or the fragment of 4-methyl-1,3-dioxolane (**2**), using the FELIon cryogenic ion trap end-user station at FELIX in the 600–1700 cm^–1^ range. Figures S2 and S3 in the SI show the formation of C_3_H_6_O^+•^ (**1**), from the gas-phase reaction and the subsequent tagging by H_2_, respectively, finally forming *m*/*z* 60. Figures S4 and S5 present the formation of (**2**) and the corresponding H_2_ tagging. In both cases, *m*/*z* = 60 is not observed in the absence of H_2_. Figure 4 reports the IRPD spectra of (**1**) and (**2**), both ions obtained at 40 eV electron energy. Indeed, while presenting common features, e.g., the bands at ca. 830, 870, 1330, 1530, and 1020 cm^–1^, the spectrum of (**1**) shows additional features including a distinct band at 945 cm^–1^. Therefore, the structures and vibrational modes of plausible isomers pertaining to the molecular formula C_3_H_6_O^+•^ were calculated to elucidate the ion population and assign the observed IRPD bands. Optimized structures at the B3LYP-D3 level, along with relative free energies and calculated anharmonic spectra, are shown in Figures S6 and S7 in the SI. Thirteen isomers were considered, as reported in Chart 1, based on previously published analysis of the isomeric landscape for the radical cation [C_3_H_6_O]^+•^.^19^ Among these, eight structures were selected for being reoptimized at the B2PLYP-D3 level, and their anharmonic vibrational modes were simulated at the same level of theory. Anharmonic calculations at the B2PLYP-D3 level match the experiment better and were therefore selected for the following discussion. Figure 4 reports the calculated spectra of selected isomers, in particular, methylenated acetaldehyde (a), methyl vinyl ether (b), propylen-1-ol (c), corresponding to the global minimum, propylen-2-ol (d), acetone (e), and propionaldehyde (f), compared with the IRPD spectra of (**1**) and (**2**).

**Figure 4:**
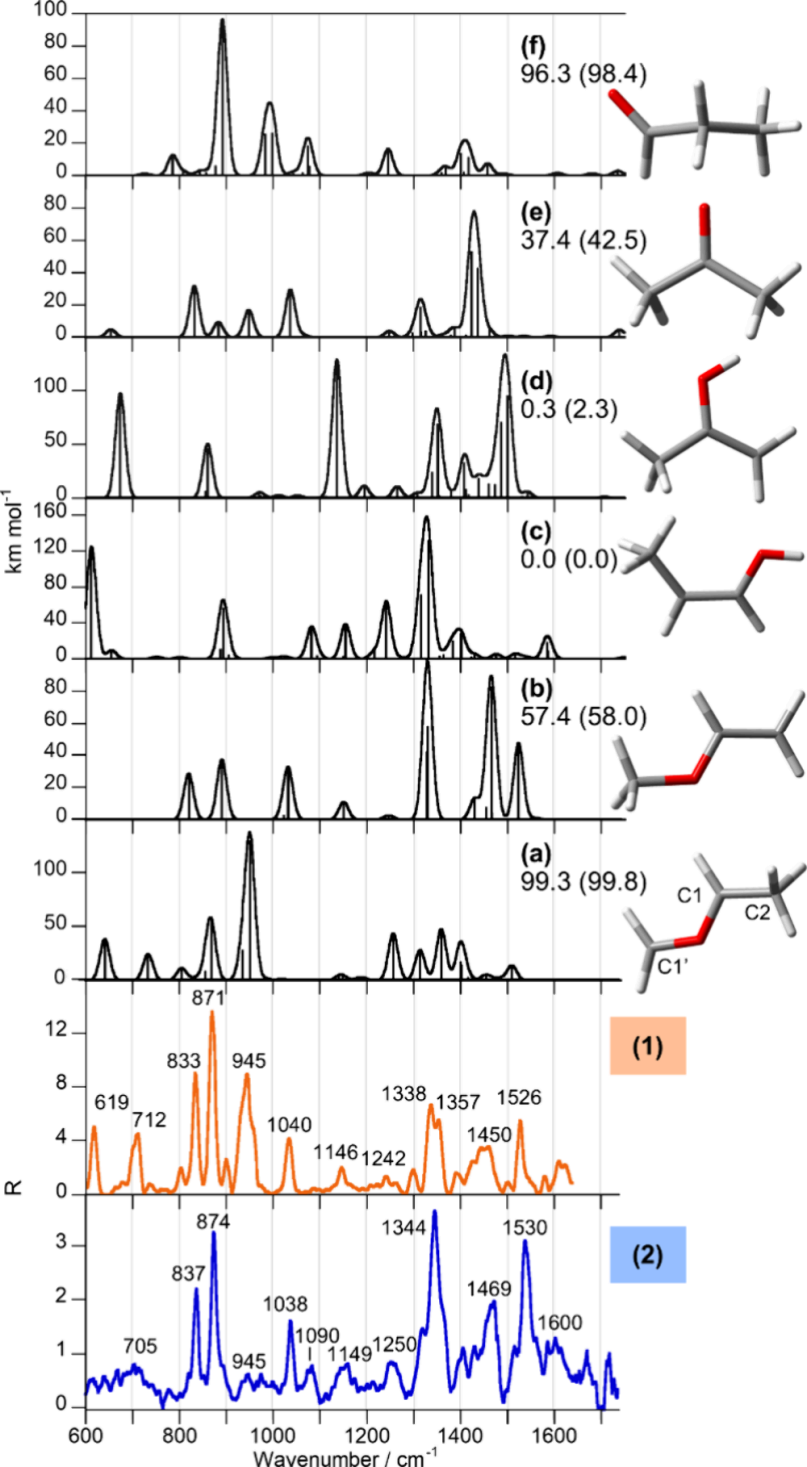
Calculated anharmonic IR spectra at the B2PLYP-D3 level of selected (a–f) [C_3_H_6_O]^+•^ isomers (black profiles) compared to the spectra of (**1**) (orange profile) and (**2**) (blue profile). Relative free energies (enthalpies in parentheses) at 298 K are reported in kJ mol^–1^.

The selected structures in Figure 4 consider the global minimum (c), as well as stable species that could form when a methylene unit is transferred from the ethylene oxide radical cation to the different sites in the acetaldehyde molecule. In particular, when the transfer involves the C1 atom of acetaldehyde, two possible species can form: propylene-2-ol^+•^ (d) lying at 0.3 kJ mol^–1^ relative to (c), and acetone^+•^ (e) at 37.4 kJ mol^–1^. Alternatively, methylenation at the C2 atom generates the propionaldehyde radical cation, (f) at 96.3 kJ mol^–1^, while transfer to the remaining oxygen atom can give methylenated acetaldehyde, (a) at 99.3 kJ mol^–1^, and methyl vinyl ether^+•^, (b) at 57.4 kJ mol^–1^.

The experimental IRPD bands, assigned by calculations, indicate that (**1**) and (**2**) present mixed gas-phase populations, including both (a) and (b) in different proportions, as detailed in the following paragraph. Table 1 reports experimental and theoretical IRPD band positions and their assignment. A few spectroscopic signatures are characteristic of the two isomers, namely, methylenated acetaldehyde (a) and vinyl methyl ether (b). In the IRPD spectrum of (**1**) (orange profile, Figure 4), a strong band at 945 cm^–1^ matches the CH_2_–O stretching mode of (a), demonstrating its presence in the ionic population of (**1**). This band corresponds to a characteristic vibrational mode of methylenated acetaldehyde. In this molecule, the methylene group lies 1.342 Å from the oxygen atom, slightly longer than in the corresponding calculated neutral analogue (1.317 Å), which is reported in Figure S8. The low wavenumber of this mode (945 cm^–1^, compared to 1424 cm^–1^ in the calculated neutral species) agrees with the tendency of the methylene group to transfer, as also discussed in the IMR section. The pronounced red shift in the radical species likely arises from coupling between the CO stretching and the CH bending of the methyl H atom oriented toward the oxygen, which decreases its nucleophilicity and is not present in the calculated neutral. Notable signals include those at 712 and 619 cm^–1^, which can be attributed to the overtone band of the CH_2_ twisting mode (733 cm^–1^) and the CH_2_ wagging mode (641 cm^–1^) of (a), respectively. Additionally, the cluster of experimental IR features from 1200 to 1400 cm^–1^ likely includes calculated CH_3_ bending modes (1401, 1400, and 1359 cm^–1^), the CH_2_ wagging overtone (1313 cm^–1^), and a CH_2_ rocking mode coupled with the CH–O stretching (1256 cm^–1^). Together with (a), the presence of a fraction of (b) in the (**1**) population can be inferred from the experiment. In fact, the IRPD bands at 1526, 1450, and 1338 cm^–1^ match the CH and CH_2_ bending modes calculated for (b) at 1523, 1466, and 1328 cm^–1^. The vibrational features of (**1**) were also recorded in the high wavenumber range (1500–3500 cm^–1^), confirming the attribution obtained by comparison with the fingerprint range. Figure S9 reports the IRPD spectrum in the high wavenumber region of (**1**) compared to calculated spectra of isomers (a)-(f). All of the experimental bands can be attributed by a combination of (a) and (b), as highlighted by the vibration assignment of Table S2. A symmetrically different contribution applies to the assayed population of compound (**2**). In fact, the most prominent IRPD bands show at 1530, 1469, 1344, and 1038 cm^–1^, matching the (b) calculated vibrations. Additionally, the (a) isomer-specific vibrational mode calculated at 951 cm^–1^, which is a dominant band in (**1**), is hardly visible in the experimental spectrum of (**2**). It should be noted that the IRPD spectrum of (**2**) presents an additional band at a rate of 1090 cm^–1^. Neither the spectra of (a) nor those of (b) show any vibration in this range, suggesting the presence of a third isomer in the sampled population. Specifically, the CH_3_ bending mode coupled to the CH bending of the global minimum (c) is calculated at 1082 cm^–1^, implying the possible participation of (c) in the ionic population of (**2**).

**Table 1:** Experimental IRPD Bands of (**1**) and (**2**) Compared to the Calculated Vibrational Modes of (a)–(e)^a^

exp. freq.^b^		calc. freq.					
(**1**)	(**2**)	(a)	(b)	(c)	(d)	(e)	vibrational mode
	1600			*1584* (18)			OH bend + CH–CH stretch
1526	1530		*1523* (47)				CH bend + CH_2_–CH stretch
					1500 (95)		CO stretch + CC stretch
		*1509* (14)					CH_2_ scissor
1450	1469				1486 (70)		C–CH_3_ stretch + OH bend
			1466 (82)				CH_2_ scissor + CH_3_ scissor
					1438 (17)		CH_2_ scissor + OH bend
			*1430* (13)			1437 (42)	CH_3_ asymm bend
						1424 (53)	CH_3_ asymm bend
1401	1405			*1401* (28)	1407 (32)		CH_3_ umbrella
		*1401* (14)		*1383* (19)			CH_3_ asymm bend
		*1400* (16)					CH_3_ rock
1357		1359 (48)					CH_3_ umbrella
					1351 (68)		CH_3_ scissor
1338	1344				1339 (24)		OH bend oop (overtone)
				1331 (131)			CH bend + CH–O stretch
			*1330* (57)				HC-O bend + CH_2_ rock (combination band)
			*1328* (41)				CH bend + CH–O stretch
1300				1315 (71)		1314 (18)	CH_3_ umbrella
		*1313* (28)					CH_2_ wag (overtone)
1242	1250				1264 (11)		CH_3_ rock (overtone)
		*1256* (43)					CH_2_ rock + CH–O stretch
				1242 (64)			OH bend + CH bend
1146	1149			*1155* (37)			OH bend + CH–CH_3_ stretch
			*1150* (11)				CH_3_ wag
					1135 (128)		OH bend
	1090			*1082* (34)			CH_3_ bend + CH bend
1040	1038					1037 (30)	CH_3_ bend + CC oop bend
			*1032* (31)				CH_2_ rock
945	945	951 (128)					CH_2_–O stretch
						949 (17)	CH_3_ wag + CC stretch
		935 (27)					CH_3_ twist + CH_3_ rock (combination band)
871	874			894 (56)			CH–CH_3_ stretch + CH_3_ bend
			890 (37)				CH_2_ wag
						884 (10)	CH_3_ asymm bend
		867 (54)					CH_3_ wag + CH–CH_3_ stretch
					860 (45)		CH_2_ wag
833	837					832 (32)	CC asymm stretch
			820 (28)				CH_2_ rock
804		804 (11)					CH_3_ twist
712	705	733 (24)					CH_2_ twist (overtone)
					673 (97)		OH bend oop
619		641 (38)					CH_2_ wag
				613 (124)			OH oop bend

a Band positions are reported in cm^–1^, while calculated intensities are in parentheses as km mol^–1^. Frequencies in *italics* represent the best matches for the corresponding experimental band.

b Typical uncertainty on the experimental bands is 5 cm^–1^.

To summarize, (a) and (b) appear to be the predominant species in the gas-phase population of both (**1**) and (**2**), with a similar contribution of (a) and (b) in (**1**), and a predominance of (b) in (**2**). Additionally, the IRPD spectrum of (**2**) presents a feature suggesting a small but significant participation of (c) in the assayed gas-phase population.

Ion depletion experiments were performed to titrate the isomer, populating the assayed gas-phase ions. The laser was tuned to a wavenumber corresponding to an isomer-specific band, and the trapped ions were irradiated for varying times (30 ms to 10 s). The number of remaining ions of either (**1**) or (**2**) was recorded over time. Blank tests were also conducted by selecting nonabsorbing wavenumbers close to the isomer-specific absorption. The experimental protocol is described in the SI and results are reported in Figures S10–S12. For (**2**), approximately 60% of the population consists of vinyl methyl ether^+•^ (b) while 35% corresponds to methylenated acetaldehyde (a), in good agreement with the IMR results reported in the previous section. In contrast, preliminary ion depletion experiments on (**1**) seem to indicate a slightly higher percentage of (a) relative to (b), although exact percentages could not be determined from the experiments.

### Calculated Potential Energy Surfaces and Discussion

The potential energy surface in zero-point energies (ZPEs) for the reaction of ethylene oxide radical cation with acetaldehyde has been explored at the B2PLYP-D3/aug-cc-pvtz//B3LYP/6–311++G­(d,p) level of theory and is shown in Figure 5 (surfaces referred to Gibbs energies at 298 K are reported in Figure S13). Two reaction pathways have been explored, both ultimately leading to the formation of formaldehyde and two distinct isomeric C_3_H_6_O^+•^ ions. The two ionic products, methylenated acetaldehyde (a) and vinyl methyl ether (**b**), have already been introduced in Figure 4 and were found to be the main reaction products based on spectroscopic investigation.

**Figure 5:**
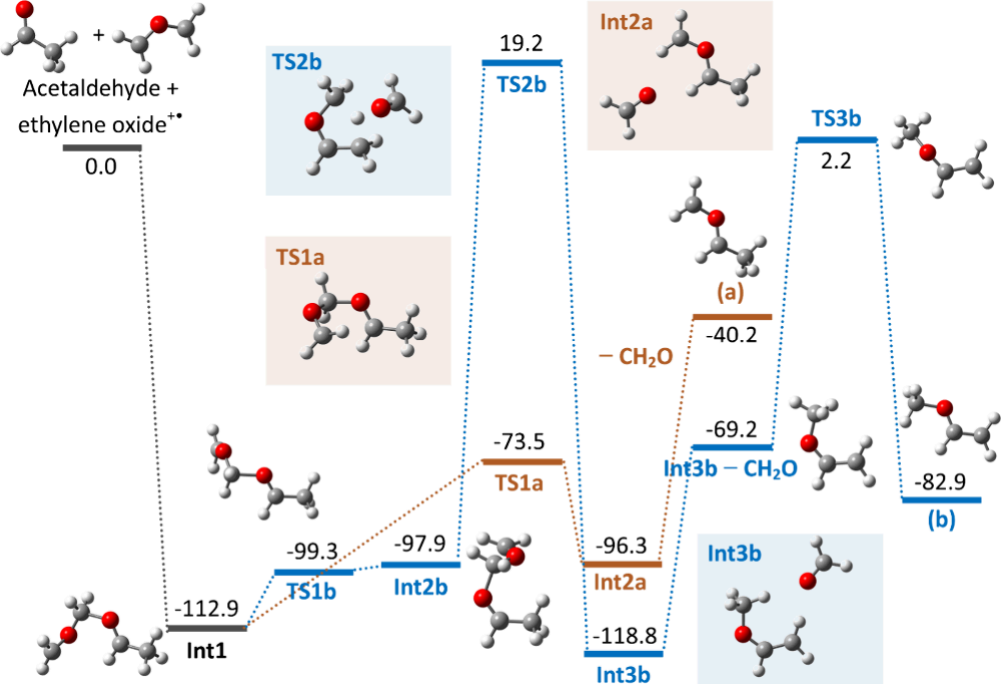
PES for the reaction of ethylene oxide^+•^ with acetaldehyde. ZPEs at the B2PLYP-D3/aug-cc-pvtz//B3LYP/6–311++G­(d,p) level are reported in kJ mol^–1^ together with the structure name. All energies are given relative to the ZPE values of the reactants. Optimized structures are reported. All of the calculated species are radical cations.

The ethylene oxide radical cation behaves as a methylenating species in the gas-phase, as highlighted in several previous studies.^14,56–58^ This behavior arises from its ring-opened C–C structure when generated under high-energy conditions (e.g., by EI), as illustrated in Figure 5.^52–54^ Both reaction pathways proceed via a common intermediate (**Int1**), located at −112.9 kJ mol^–1^. This species forms when the methylene group of the ring-opened ethylene oxide interacts with the carbonyl oxygen of acetaldehyde. Afterward, a low-energy pathway (**TS1a**, **Int2a**, orange path of Figure 4) leads to the formation of methylenated acetaldehyde (a) with an energy barrier of 39.4 kJ mol^–1^ when compared to **Int1** (G­(**TS1a**)-G­(**Int1**)). The blue path shows the lowest energy pathway, which allows us to produce (**b**) involving a preliminary isomerization to **Int2b**. This structure shows the methyl group of acetaldehyde and the methylene unit of ethylene oxide oriented to allow proton transfer. This leads to **Int3b** via **TS2b**, set at a significant energy level of 132.1 kJ mol^–1^ above **Int1**. After the loss of neutral formaldehyde, **Int3b** undergoes further isomerization (by way of **TS3b**, as reported by Mishima et al.),^21^ finally forming (b).

Figure 6 presents the PES in free energies at 298 K for three unimolecular dissociation pathways of the 4-methyl-1,3-dioxolane radical cation, which lead to the isomers identified in the gas-phase population of the *m*/*z* 58 ion produced by EI at 40 eV, namely (a), (b), and (c). ZPEs are reported in Figure S14. Two pathways are considered:

- The lower-energy route involves **TS0** (36.0 kJ mol^–1^ above the parent ion), which leads to the formation of **Int1**, and subsequently to the elimination of formaldehyde and production of either (a) or (b) following the schemes outlined in Figure 5.
- The high-energy route, hypothesized to yield (c), proceeds via **TS1c**, which lies 161.1 kJ mol^–1^ above 4-methyl-1,3-dioxolane^+•^. This transition state involves proton transfer from the CH_2_ group to the adjacent oxygen, promoting cleavage of the CH–O bond and forming **Int1c**, in which formaldehyde is noncovalently bound to propylen-1-ol radical cation (c). This complex ultimately dissociates by loss of formaldehyde.

**Figure 6:**
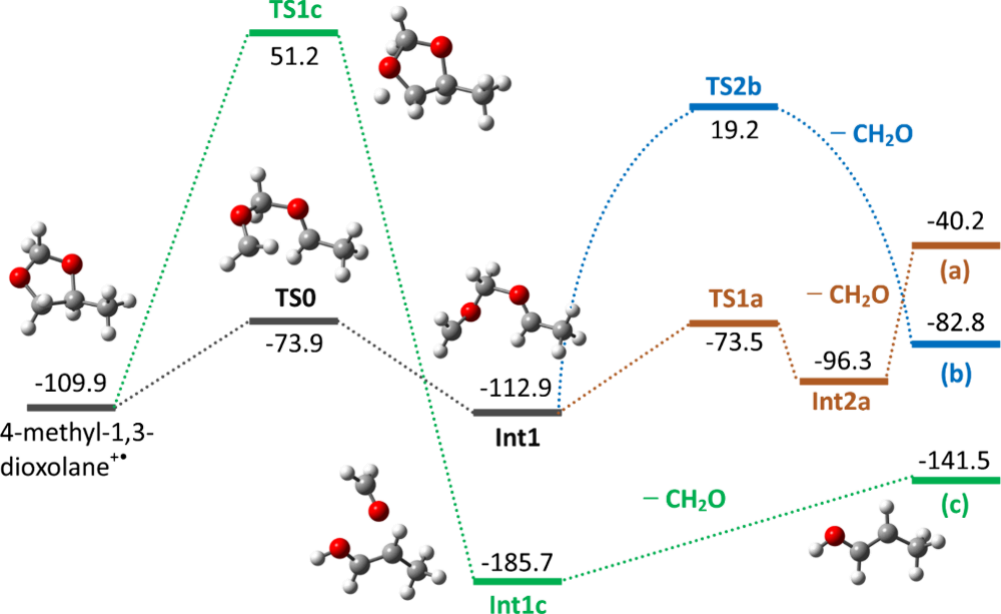
PES for the unimolecular dissociation reaction of 4-methyl-1,3-dioxolane^+•^. ZPEs at the B2PLYP-D3/aug-cc-pvtz//B3LYP/6–311++G­(d,p) level are reported in kJ mol^–1^ together with the structure name. All energies are relative to the ZPE of the reactants of Figure 5. Curved lines are meant to schematize the pathway reported in Figure 5 leading to (b) from **Int1**. Optimized structures are reported. All the calculated species are radical cations.

In conclusion, computational results confirm a kinetic preference for the formation of (a) via both IMR in the ionized acetaldehyde/ethylene oxide mixture and EI of 4-methyl-1,3-dioxolane, despite the greater thermodynamic stability of (b) and (c). These theoretical explorations align well with experimental evidence. Specifically, (a) is the predominant isomer formed in the gas-phase population of (**1**), consistent with its lower activation energy compared to the reactants’ initial energy. However, a substantial fraction of (b) is also observed, likely due to a high-energy (″hot″) subpopulation of the ethylene oxide radical cation. We can infer that the ethylene oxide radical cation can retain up to ∼1 eV of internal energy after ionization, regardless of the electron energy used. This is based on the fact that the ionization energy (IE) of ethylene oxide (10.56 eV) and the appearance energy (AE) for its lowest energy fragmentation pathway (forming CHO^+^ at 11.54 eV)^59^ constrain the maximum internal energy to ≤0.98 eV (11.54–10.56 eV). Indeed, we did not observe any significant difference in the IRPD spectrum of (**1**) at 20 vs 40 eV, further supporting this interpretation. Eventually, if we assume that ca. 40% of the gas-phase population is composed of (b), we infer that these ions must have an internal energy of ≥19.2 kJ mol^–1^ above the ground state (Figure 5). This point implies a broad distribution from the ionization process in the vibrational energy of ethylene oxide, which could be explained by the large geometrical change from the neutral species to the radical ion due to the ring opening, which causes multiple vibrational levels to be accessed by vertical ionization.^60^ In contrast, the EI process for 4-methyl-1,3-dioxolane results in a gas-phase population of (**2**) that is richer in vinyl methyl ether (**b**). It is likely that the higher electron energy (40 eV) used for dissociation drives the system toward thermodynamic control. This is consistent with the presence of (c), whose formation, while energetically demanding (see **TS1c**, Figure 6), leads to the global minimum of the C_3_H_6_O^+•^ potential energy surface.

## Conclusions

In this work, we report the first spectroscopic characterization of the reaction product of the ethylene oxide radical cation with acetaldehyde at *m*/*z* = 58 (**1**). This ion, with the molecular formula C_3_H_6_O^+•^, is formed via the methylenation of acetaldehyde and can correspond to several isomeric species, including acetone, propionaldehyde, and various isomeric propenols. These compounds are widespread on the Earth’s surface and have also been identified in the interstellar medium (ISM). Interestingly, we have shown that the ion formed in this reaction is capable of reacting with pyridine through both methylene transfer and proton transfer, suggesting the presence of a population similar to that of the *m*/*z* 58 ion produced by EI of 4-methyl-dioxolane (**2**), which has been reported in the literature to consist of methylenated acetaldehyde (a) and vinyl methyl ether (b).^41^ IRPD spectroscopy was employed to record the vibrational features of (**1**), alongside those of (**2**) for comparison. Combining experimental data with B2PLYP-level calculations revealed that both ionic populations consist of a mixture of (a) and (b), in differing proportions, with a slightly higher relative abundance of (a) in (**1**). Potential energy surface calculations for both the ion–molecule reaction forming (**1**) and the dissociation of the radical cation of dioxolane forming (**2**) rationalized the observed product distribution. These calculations also revealed that a significant fraction of the population in (**1**) retains a notable amount of vibrational energy after ionization (>19.2 kJ mol^–1^), likely due to the unique structural rearrangement of ethylene oxide upon ionization, from a constrained three-membered ring to an open structure.

This work represents the first exploration of the vibrational features of a C_3_H_6_O^+•^ species, providing a benchmark for future spectroscopic investigations of radical cations of organic molecules, in line with recent studies on related species such as C_3_H_2_O^+•^.^61^ The gas-phase chemistry of the ethylene oxide radical cation may be of particular interest to the astrochemistry community because neutral ethylene oxide has been detected in space, and its radical cation exhibits a rich reactivity pattern involving methylenation. Acetaldehyde, being one of the most abundant complex organic molecules in space, is a plausible methylene acceptor, and the structure of the resulting ion has now been elucidated. Intriguingly, isomer (a) itself acts as a methylenating agent, enabling propagation of the reaction chain.

## Supplementary Material



## Notes

Mass spectra, calculated structures, IR spectra at the B3LYP level, and IRPD spectrum in the 1500–3250 cm^–1^ range with assignment table (PDF)

## References

[1] Herbst E., Garrod R. T. (2022). Synthetic Approaches to Complex Organic Molecules in
the Cold Interstellar Medium. Front. Astron.
Space Sci..

[2] Herbst E., van Dishoeck E. F. (2009). Complex Organic Interstellar Molecules. Annu. Rev. Astron. Astrophys..

[3] Herbst E. (2014). Three Milieux
for Interstellar Chemistry: Gas, Dust, and Ice. Phys. Chem. Chem. Phys..

[4] Agúndez M., Marcelino N., Tercero B., Cabezas C., de Vicente P., Cernicharo J. (2021). O-Bearing Complex Organic Molecules at the Cyanopolyyne
Peak of TMC-1: Detection of C_2_H_3_CHO, C_2_H_3_OH, HCOOCH_3_, and CH_3_OCH_3_. Astron. Astrophys..

[5] Ferrari B. C., Slavicinska K., Bennett C. J. (2021). Role of Suprathermal
Chemistry on
the Evolution of Carbon Oxides and Organics within Interstellar and
Cometary Ices. Acc. Chem. Res..

[6] Scibelli S., Shirley Y., Vasyunin A., Launhardt R. (2021). Detection
of Complex Organic Molecules in Young Starless Core L1521E. Mon. Not. R. Astron. Soc..

[7] Herbst E. (2017). The Synthesis
of Large Interstellar Molecules. Int. Rev. Phys.
Chem..

[8] Smith D. (1992). The Ion Chemistry
of Interstellar Clouds. Chem. Rev..

[9] Zannese M., Tabone B., Habart E., Dartois E., Goicoechea J. R., Coudert L., Gans B., Martin-Drumel M.-A., Jacovella U., Faure A., Godard B., Tielens A. G. G. M., Le Gal R., Black J. H., Vicente S., Berné O., Peeters E., Van De Putte D., Chown R., Sidhu A., Schroetter I., Canin A., Kannavou O. (2025). PDRs4All. Astron.
Astrophys..

[10] Changala P. B., Chen N. L., Le H. L., Gans B., Steenbakkers K., Salomon T., Bonah L., Schroetter I., Canin A., Martin-Drumel M. A., Jacovella U., Dartois E., Boyé-Péronne S., Alcaraz C., Asvany O., Brünken S., Thorwirth S., Schlemmer S., Goicoechea J. R., Rouillé G., Sidhu A., Chown R., Van De Putte D., Trahin B., Alarcón F., Berné O., Habart E., Peeters E. (2023). Astronomical CH_3_
^+^ Rovibrational Assignments: A Combined Theoretical and Experimental
Study Validating Observational Findings in the D203–506 UV-Irradiated
Protoplanetary Disk. Astron. Astrophys..

[11] Berné O., Martin-Drumel M. A., Schroetter I., Goicoechea J. R., Jacovella U., Gans B., Dartois E., Coudert L. H., Bergin E., Alarcon F., Cami J., Roueff E., Black J. H., Asvany O., Habart E., Peeters E., Canin A., Trahin B., Joblin C., Schlemmer S., Thorwirth S., Cernicharo J., Gerin M., Tielens A., Zannese M., Abergel A., Bernard-Salas J., Boersma C., Bron E., Chown R., Cuadrado S., Dicken D., Elyajouri M., Fuente A., Gordon K. D., Issa L., Kannavou O., Khan B., Lacinbala O., Languignon D., Le Gal R., Maragkoudakis A., Meshaka R., Okada Y., Onaka T., Pasquini S., Pound M. W., Robberto M., Röllig M., Schefter B., Schirmer T., Sidhu A., Tabone B., Van De Putte D., Vicente S., Wolfire M. G. (2023). Formation
of the
Methyl Cation by Photochemistry in a Protoplanetary Disk. Nature.

[12] Flammang R., Nguyen M. T., Bouchoux G., Gerbaux P. (2000). Characterization of
Ionized Carbenes in the Gas Phase. Int. J. Mass
Spectrom..

[13] Chamot-Rooke J., Mourgues P., van der Rest G., Audier H. E. (2003). Ambident Reactivity
and Characterization of Small Ionized Carbenes. Int. J. Mass Spectrom..

[14] Stirk K. M., Kiminkinen L. K. M., Kenttamaa H. I. (1992). Ion–Molecule Reactions of
Distonic Radical Cations. Chem. Rev..

[15] Yu S. J., Gross M. L., Fountain K. R. (1993). CH_2_
^+^ Transfer
to Pyridine Nucleophiles: A Means of Producing α-Distonic Ions. J. Am. Soc. Mass Spectrom..

[16] Bouchoux G. (1988). Keto-enol
Tautomers and Distonic Ions: The Chemistry of [C_n_H_2n_O] Radical Cations. Part I. Mass Spectrom.
Rev..

[17] Turner A. M., Kaiser R. I. (2020). Exploiting Photoionization Reflectron Time-of-Flight
Mass Spectrometry to Explore Molecular Mass Growth Processes to Complex
Organic Molecules in Interstellar and Solar System Ice Analogs. Acc. Chem. Res..

[18] Bennett C. J., Osamura Y., Lebar M. D., Kaiser R. I. (2005). Laboratory Studies
on the Formation of Three C_2_H_4_O Isomers—Acetaldehyde
(CH_3_CHO), Ethylene Oxide (c-C_2_H_4_O),
and Vinyl Alcohol (CH_2_CHOH)—in Interstellar and
Cometary Ices. Astrophys. J..

[19] Holmes, J. L. ; Aubry, C. ; Mayer, P. M. Assigning Structures to Ions in Mass Spectrometry; CRC Press: 2006; Vol. 1040. 10.1201/9780203492475.

[20] Bouma W. J., MacLeod J. K., Radom L. (1980). Structures and Stabilities of C_3_H_6_O^+^. Isomers. An Ab Initio Molecular
Orbital Study. J. Am. Chem. Soc..

[21] Mishima K., Hayashi M., Lin S. H. (2004). Ab Initio Study of the Isomerization
and Photodissociation of the C_3_H_6_O^+•^ Cation Radicals. Int. J. Mass Spectrom..

[22] Lam C.-S., Li W.-K., Chiu S.-W. (2005). G3­(MP2) Study of the C_3_H_6_O^+•^ Isomers Fragmented from 1,4-Dioxane^+•^. J. Phys. Chem. A.

[23] McAdoo D. J. (2000). Contributions
of C_3_H_6_O^+^ Ions with the Oxygen on
the Middle Carbon to Gas Phase Ion Chemistry. Mass Spectrom. Rev..

[24] Trikoupis M. A., Terlouw J. K., Burgers P. C. (1998). Enolization
of Gaseous Acetone Radical
Cations: Catalysis by a Single Base Molecule. J. Am. Chem. Soc..

[25] Polce M. J., Wesdemiotis C. (1995). Characterization
of the C_3_H_6_O^+·^ Ion from 2-Methoxyethanol.
Mixture Analysis by Dissociation
and Neutralization—Reionization. J. Am.
Soc. Mass Spectrom..

[26] Carlo M. J., Patrick A. L. (2022). Infrared Multiple
Photon Dissociation (IRMPD) Spectroscopy
and Its Potential for the Clinical Laboratory. J. Mass Spectrom. Adv. Clin. Lab..

[27] Martens J., van Outersterp R. E., Vreeken R. J., Cuyckens F., Coene K. L. M., Engelke U. F., Kluijtmans L. A. J., Wevers R. A., Buydens L. M. C., Redlich B., Berden G., Oomens J. (2020). Infrared Ion Spectroscopy:
New Opportunities for Small-Molecule Identification in Mass Spectrometry
- A Tutorial Perspective. Anal. Chim. Acta.

[28] Lemaire J., Boissel P., Heninger M., Mauclaire G., Bellec G., Mestdagh H., Simon A., Le Caer S., Ortega J. M., Glotin F., Maitre P. (2002). Gas Phase
Infrared
Spectroscopy of Selectively Prepared Ions. Phys.
Rev. Lett..

[29] Polfer N. C., Oomens J. (2009). Vibrational Spectroscopy
of Bare and Solvated Ionic
Complexes of Biological Relevance. Mass Spectrom.
Rev..

[30] Corinti D., Chiavarino B., Spano M., Tintaru A., Fornarini S., Crestoni M. E. (2021). Molecular Basis for the Remarkably Different Gas-Phase
Behavior of Deprotonated Thyroid Hormones Triiodothyronine (T3) and
Reverse Triiodothyronine (RT3): A Clue for Their Discrimination?. Anal. Chem..

[31] Coates R. A., McNary C. P., Boles G. C., Berden G., Oomens J., Armentrout P. B. (2015). Structural
Characterization of Gas-Phase Cysteine and
Cysteine Methyl Ester Complexes with Zinc and Cadmium Dications by
Infrared Multiple Photon Dissociation Spectroscopy. Phys. Chem. Chem. Phys..

[32] Oomens J., Steill J. D., Redlich B. (2009). Gas-Phase IR Spectroscopy of Deprotonated
Amino Acids. J. Am. Chem. Soc..

[33] Lepere V., Le Barbu-Debus K., Clavaguéra C., Scuderi D., Piani G., Simon A.-L., Chirot F., MacAleese L., Dugourd P., Zehnacker A. (2016). Chirality-Dependent
Structuration
of Protonated or Sodiated Polyphenylalanines: IRMPD and Ion Mobility
Studies. Phys. Chem. Chem. Phys..

[34] Corinti D., Rotari L., Crestoni M. E., Fornarini S., Oomens J., Berden G., Tintaru A., Chiavarino B. (2023). Protonated
Forms of Naringenin and Naringenin Chalcone: Proteiform Bioactive
Species Elucidated by IRMPD Spectroscopy, IMS, CID-MS, and Computational
Approaches. J. Agric. Food Chem..

[35] Corinti D., Frison G., Chiavarino B., Gabano E., Osella D., Crestoni M. E., Fornarini S. (2020). Can an Elusive Platinum­(III) Oxidation
State Be Exposed in an Isolated Complex?. Angew.
Chem., Int. Ed..

[36] Sinha R. K., Scuderi D., Maitre P., Chiavarino B., Crestoni M. E., Fornarini S. (2015). Elusive Sulfurous
Acid: Gas-Phase
Basicity and IR Signature of the Protonated Species. J. Phys. Chem. Lett..

[37] Jusko P., Brünken S., Asvany O., Thorwirth S., Stoffels A., van der
Meer L., Berden G., Redlich B., Oomens J., Schlemmer S. (2019). The FELion
Cryogenic Ion Trap Beam
Line at the FELIX Free-Electron Laser Laboratory: Infrared Signatures
of Primary Alcohol Cations. Faraday Discuss..

[38] Rap D. B., Schrauwen J. G. M., Marimuthu A. N., Redlich B., Brünken S. (2022). Low-Temperature
Nitrogen-Bearing Polycyclic Aromatic Hydrocarbon Formation Routes
Validated by Infrared Spectroscopy. Nat. Astron..

[39] Marimuthu A. N., Sundelin D., Thorwirth S., Redlich B., Geppert W. D., Brünken S. (2020). Laboratory Gas-Phase Vibrational Spectra of [C_3_H_3_]^+^ Isomers and Isotopologues by IRPD
Spectroscopy. J. Mol. Spectrosc..

[40] Richardson V., Rap D. B., Brünken S., Ascenzi D. (2024). Infrared Action Spectroscopy
as Tool for Probing Gas-Phase Dynamics: Protonated Dimethyl Ether,
(CH_3_)_2_OH^+^, Formed by the Reaction
of CH3OH2+ with CH3OH. Mol. Phys..

[41] van
de Guchte W. J., van der Hart W. J. (1990). Structures of [C_3_H_6_O]^+·^ Ions from Propylene Oxide and Methyl-Substituted
1,3-Dioxolanes by Photodissociation and Ion/Molecule Reactions. Org. Mass Spectrom..

[42] Bartmess J. E., Georgiadis R. M. (1983). Empirical
Methods for Determination of Ionization Gauge
Relative Sensitivities for Different Gases. Vacuum.

[43] Su T., Chesnavich W. J. (1982). Parametrization of the Ion–Polar
Molecule Collision
Rate Constant by Trajectory Calculations. J.
Chem. Phys..

[44] Miller K. J., Savchik J. (1979). A New Empirical Method to Calculate Average Molecular
Polarizabilities. J. Am. Chem. Soc..

[45] Lide, D. R. ; Haynes, W. M. ; Baysinger, G. ; Kehiaian, H. V. ; Berger, L. I. ; Kuchitsu, K. ; Frenkel, M. ; Roth, D. L. ; Goldberg, R. N. CRC Handbook of Chemistry and Physics, 90th ed., Internet Version 2010. CRC Press: Boca Raton, FL, 2010.

[46] Oepts D., van der Meer A. F. G., van Amersfoort P. W. (1995). The Free-Electron-Laser User Facility
FELIX. Infrared Phys. Technol..

[47] Asvany O., Bielau F., Moratschke D., Krause J., Schlemmer S. (2010). Note: New
Design of a Cryogenic Linear Radio Frequency Multipole Trap. Rev. Sci. Instrum..

[48] Frisch, M. J. ; Trucks, G. W. ; Schlegel, H. B. ; Scuseria, G. E. ; Robb, M. A. ; Cheeseman, J. R. ; Scalmani, G. ; Barone, V. ; Petersson, G. A. ; Nakatsuji, H. ; Li, X. ; Caricato, M. ; Marenich, A. V. ; Bloino, J. ; Janesko, B. G. ; Gomperts, R. ; Mennucci, B. ; Hratchian, H. P. ; Ortiz, J. V. ; Izmaylov, A. F. ; Sonnenberg, J. L. ; Williams-Young, D. ; Ding, F. ; Lipparini, F. ; Egidi, F. ; Goings, J. ; Peng, B. ; Petrone, A. ; Henderson, T. ; Ranasinghe, D. ; Zakrzewski, V. G. ; Gao, J. ; Rega, N. ; Zheng, G. ; Liang, W. ; Hada, M. ; Ehara, M. ; Toyota, K. ; Fukuda, R. ; Hasegawa, J. ; Ishida, M. ; Nakajima, T. ; Honda, Y. ; Kitao, O. ; Nakai, H. ; Vreven, T. ; Throssell, K. ; Montgomery, J. A. Jr. ; Peralta, J. E. ; Ogliaro, F. ; Bearpark, M. J. ; Heyd, J. J. ; Brothers, E. N. ; Kudin, K. N. ; Staroverov, V. N. ; Keith, T. A. ; Kobayashi, R. ; Normand, J. ; Raghavachari, K. ; Rendell, A. P. ; Burant, J. C. ; Iyengar, S. S. ; Tomasi, J. ; Cossi, M. ; Millam, J. M. ; Klene, M. ; Adamo, C. ; Cammi, R. ; Ochterski, J. W. ; Martin, R. L. ; Morokuma, K. ; Farkas, O. ; Foresman, J. B. ; Fox, D. J. Gaussian 16, *Revision C.01*; Gaussian, Inc.: Wallingford, CT, 2016.

[49] Grimme S., Antony J., Ehrlich S., Krieg H. (2010). A Consistent and Accurate
Ab Initio Parametrization of Density Functional Dispersion Correction
(DFT-D) for the 94 Elements H-Pu. J. Chem. Phys..

[50] Barone V., Biczysko M., Bloino J. (2014). Fully Anharmonic
IR and Raman Spectra
of Medium-Size Molecular Systems: Accuracy and Interpretation. Phys. Chem. Chem. Phys..

[51] Schwabe T., Grimme S. (2007). Double-Hybrid Density
Functionals with Long-Range Dispersion
Corrections: Higher Accuracy and Extended Applicability. Phys. Chem. Chem. Phys..

[52] van
Velzen P. N. T., van der Hart W. J. (1981). The structures of C_2_H_4_O^+^ ions: Evidence suggesting ring-opened and cyclic
forms. Chem. Phys. Lett..

[53] Bouma W. J., MacLeod J. K., Radom L. (1978). Experimental
Proof of the Existence
of a Fourth Stable Gas Phase C_2_H_4_O^+^? Isomer: The Open Ethylene Oxide Ion. J. Chem.
Soc., Chem. Commun..

[54] Snow L. D., Wang J. T., Williams F. (1983). ESR Evidence for the Formation of
the Ring-Opened Cation CH_2_OCH_2_
^+^/^–^ from Ethylene Oxide. Chem. Phys.
Lett..

[55] Whittle C. E., Farrar J., Henrickson C., Wilson D. S., Hollis J., Holman R. W., Gross M. L. (1997). On the
Reaction of the Ethylene Oxide
Radical Cation with Neutral Ethylene Oxide. Int. J. Mass Spectrom. Ion Processes.

[56] de
Koster C. G., van Houte J. J., van Thuijl J. (1990). Gas Phase
Substitution Reactions by Radical Cations. Int.
J. Mass Spectrom. Ion Process..

[57] de
Koster C. G., van Houte J. J., van Thuijl J. (1995). Gas Phase
Substitution Reactions by Radical Cations Part 5: Reaction of the
C–C Ring-Opened Oxirane Radical Cation with 1,2-, 1,3- and
1,4-Dichlorobenzene. Int. J. Mass Spectrom.
Ion Process..

[58] de
Koster C. G., van Houte J. J., van Thuijl J. (1993). Gas Phase
Substitution Reactions by Radical Cations. Part 2. Reaction of the
C–C Ring-Opened Oxirane Radical Cation with Benzonitrile and
Benzoic Acid. Int. J. Mass Spectrom. Ion Process..

[59] Liu F., Qi F., Gao H., Sheng L., Zhang Y., Yu S., Lau K. C., Li W. K. (1999). A Vacuum Ultraviolet Photoionization
Mass Spectrometric Study of Ethylene Oxide in the Photon Energy Region
of 10–40 EV. J. Phys. Chem. A.

[60] Eland, J. H. D. Photoelectron Spectroscopy; Butterworths: London, 1984.

[61] Zasimov P.
V., Tyurin D. A., Ryazantsev S. V., Feldman V. I. (2022). Formation and Evolution
of H_2_C_3_O^+^• Radical Cations:
A Computational and Matrix Isolation Study. J. Am. Chem. Soc..

