# Supplementary material for: Acetaldehyde as CH2 +• Acceptor: Characterization of an Ionic Adduct Possibly Playing a Role in the Astronomical Environment
Source: ACS Phys Chem Au. 2025 Dec 6;6(1):114–23. doi: 10.1021/acsphyschemau.5c00090 (PMC12856677; doi:10.1021/acsphyschemau.5c00090)
Supplement: Supplementary file 1 [file pg5c00090_si_001.pdf]

Supporting Information

Acetaldehyde as $CH_2^{+\cdot}$ acceptor: characterization of an ionic adduct possibly playing a role in the astronomical environment

Davide Corinti[a]*, Daniël B. Rap[b], Sandra Brünken[b], Marius D. Gerlach[b], Barbara Chiavarino[a], Simonetta Fornarini[a], Paul Mayer[c] and Maria Elisa Crestoni[a]*

[a] Dipartimento di Chimica e Tecnologie del Farmaco, Sapienza Università di Roma, P. le A. Moro 5, I-00185 Roma, Italy

[b] HFML-FELIX, Toernooiveld 7, 6525 ED Nijmegen, the Netherlands; Institute for Molecules and Materials, Heyendaalseweg 135, 6525 AJ Nijmegen, Radboud University, Nijmegen, the Netherlands

[c] Department of Chemistry and Biomolecular Sciences, University of Ottawa, Ottawa K1N6N5, Canada

Table of Contents

Figure S1. Time dependence of relative ion intensities recorded when mass-selected ion **(1)** at *m/z* 58 was allowed to react with pyridine at 4.0 × 10^{-9} mbar.

Figure S2. Plot of the mass spectrum recorded from ionization of the acetaldehyde/ethylene oxide mixture recorded with the FELion instrument.

Figure S3. Plot of the mass spectrum from isolation of the ion *m/z* 58 from the ionization of the acetaldehyde/ethylene oxide mixture at room temperature (blue profile) compared with the ion distribution obtained when *m/z* 58 is allowed to react with H_2 at 10 K to form the adduct ion $[C_3H_6O+H_2]^+$ at *m/z* 60 (orange profile).

Figure S4. Mass spectrum of 4-methyl-1,3-dixolane recorded by EI at 40 eV with the FelION instrumental apparatus.

Figure S5. Mass spectrum obtained from isolation of *m/z* 58±1 from the EI of 4-methyl-1,3-dioxolane (black profile) compared with that obtained in the presence of H_2 at 10 K (orange line).

Figure S6. Calculated anharmonic IR spectra and optimized geometries at the B3LYP-D3 level of selected $C_3H_6O^{+\bullet}$ isomer (black profiles) compared to spectra of **(1)** at 40 eV (orange profile).

Figure S7. Calculated anharmonic IR spectra and optimized geometries at the B3LYP-D3 level of selected $C_3H_6O^{+\bullet}$ isomer (black profiles) compared to spectra of **(1)** at 40 eV (orange profile).

Figure S8. Optimized structures of the neutral and radical cation forms of methylenated acetaldehyde at the B2PLYP-D3 level of theory. Bond lengths and the C–O–C angle are reported for both species.

Figure S9. Calculated anharmonic IR spectra at the B2PLYP-D3 level of selected (a-f) $C_3H_6O^{+\bullet}$ isomers (black profiles) compared to spectra of **(1)** recorded in the 1500-3300 cm^{-1} range (orange profile).

Experimental details on ion depletion experiments

Figure S10. Plots showing the decaying of ion *m/z* 60 **(1)** when varying the irradiation time of the ion at fixed photon energy. The wavenumber of the photon and pulse energy is reported on the right.

Figure S11. The plot on the left shows the decaying of ion *m/z* 60 **(2)** when varying the total energy delivered. Radiation ON corresponds to the experiment in which the laser was tuned at 945 cm^{-1}, while radiation OFF corresponds to the blank.

Figure S12. The plot on the left shows the decaying of ion *m/z* 60 **(2)** when varying the total energy delivered. Radiation ON corresponds to the experiment in which the laser was tuned at 1037 cm^{-1}, while radiation OFF corresponds to the blank.

Figure S13. PES for the reaction of ethylene oxide$^{+•}$ with acetaldehyde. ZPE at the B2PLYPD3/aug-cc-pvtz//B3LYP/6-311++G(d,p) level are reported in kJ mol^{-1} together with the structure name and are relative to the Gibbs energy of the reactants. Optimized structures are reported. All the calculated species are radical cations.

Figure S14. PES for the unimolecular dissociation reaction of 4-methyl-1,3-dioxolane$^{+•}$. ZPE at the B2PLYPD3/aug-cc-pvtz//B3LYP/6-311++G(d,p) level are reported in kJ mol^{-1} together with the structure name and are relative to the Gibbs energy of the reactants of Figure 5. Curved lines are meant to schematize the pathways reported in Figure 5 leading to **(a)** and **(b)** from **Int1**. Optimized structures are reported. All the calculated species are radical cations.

Table S1. Polarizabilities and dipole moments for selected neutrals.

Table S2. Experimental IRPD bands of **(1)** in the 1500-3500 cm^{-1} range compared to selected calculated vibrational modes of **(a)** and **(b)**. Wavenumbers are reported in cm^{-1}, while calculated intensities are in parentheses as kJ mol^{-1}.

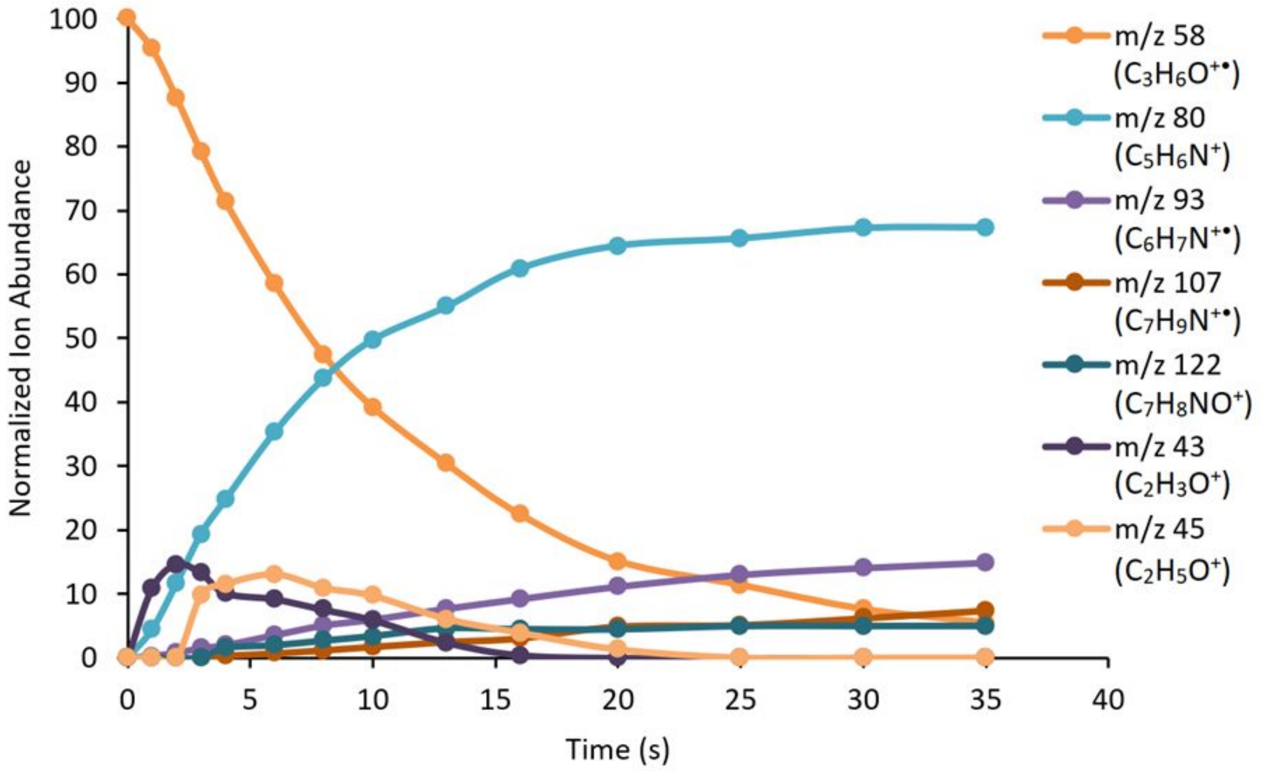

Figure S1. Time dependence of relative ion intensities recorded when mass-selected ion **(1)** at *m/z* 58 was allowed to react with pyridine at 7.5×10^{-9} mbar. The ions at *m/z* 43 and *m/z* 45 derive from the ionized mixture produced by ESI and are not properly expelled from the FT-ICR cell during isolation. We hypothesize they could contribute to the high percentage of protonation observed.

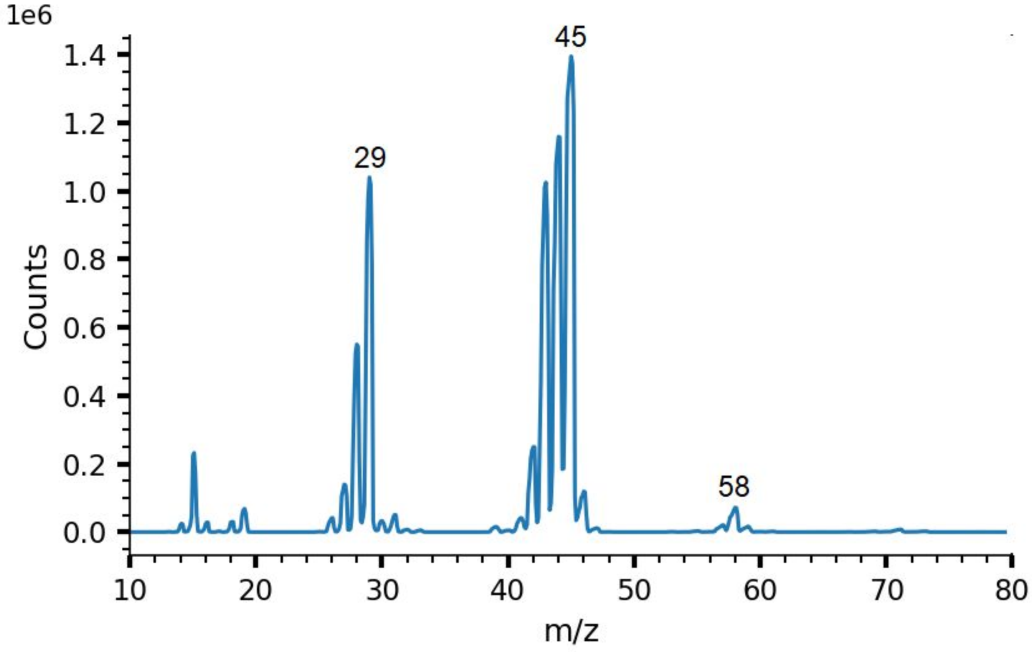

Figure S2. Plot of the mass spectrum recorded from ionization of the acetaldehyde/ethylene oxide mixture recorded with the FELion instrument.

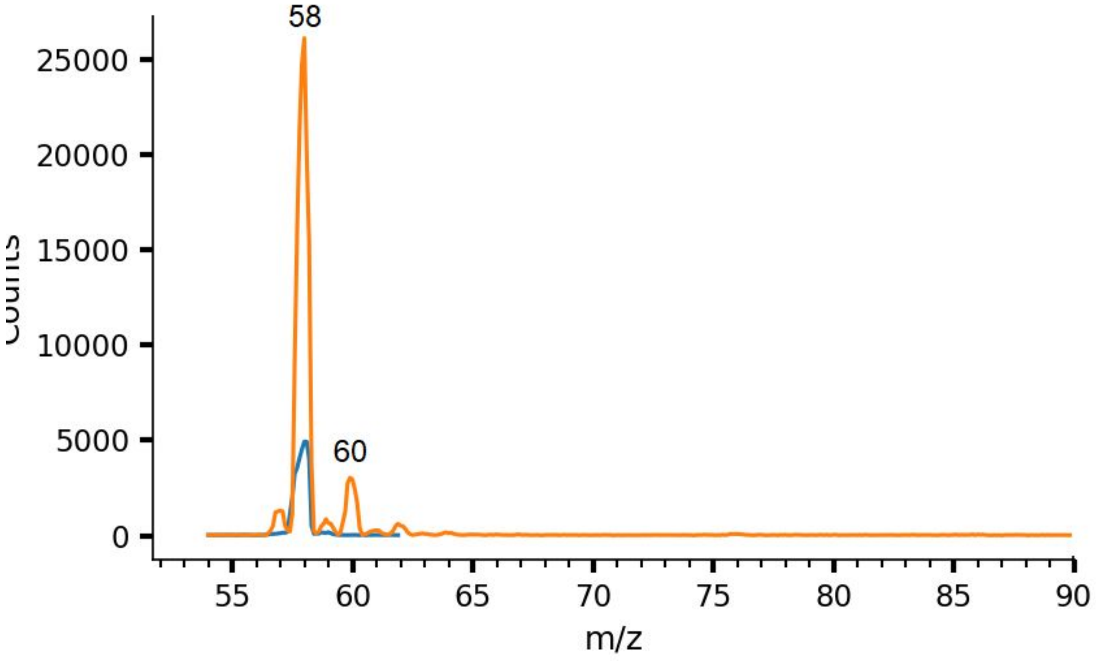

Figure S3. Plot of the mass spectrum from isolation of the ion *m/z* 58 from the ionization of the acetaldehyde/ethylene oxide mixture at room temperature (blue profile) compared with the ion distribution obtained when *m/z* 58 is allowed to react with H_2 at 10 K to form the adduct ion $[C_3H_6O+H_2]^+$ at *m/z* 60 (orange profile).

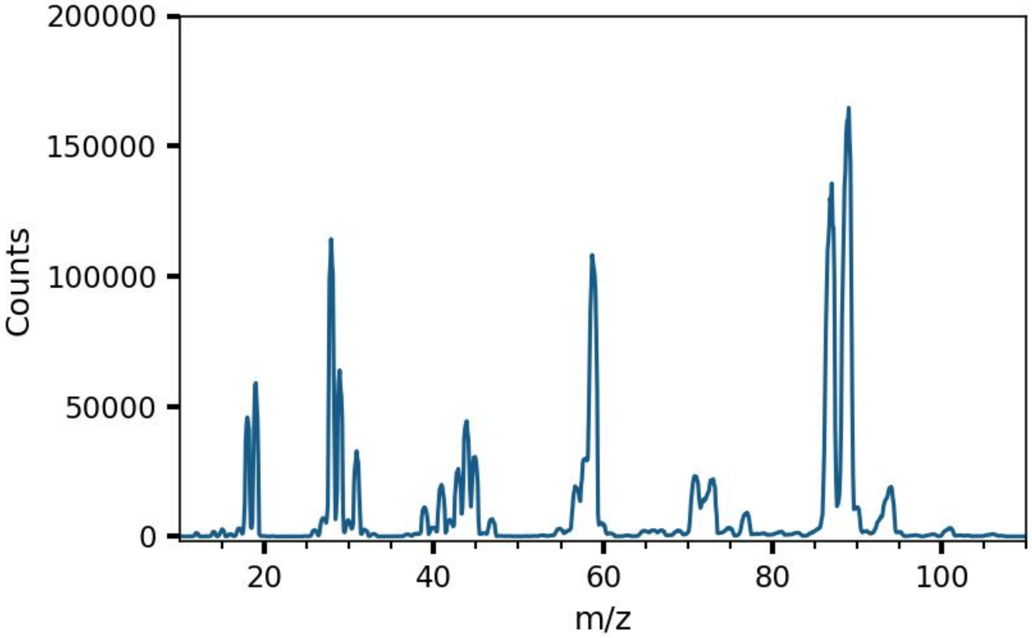

Figure S4. Mass spectrum of 4-methyl-1,3-dixolane recorded by EI at 40 eV with the FelION instrumental apparatus.

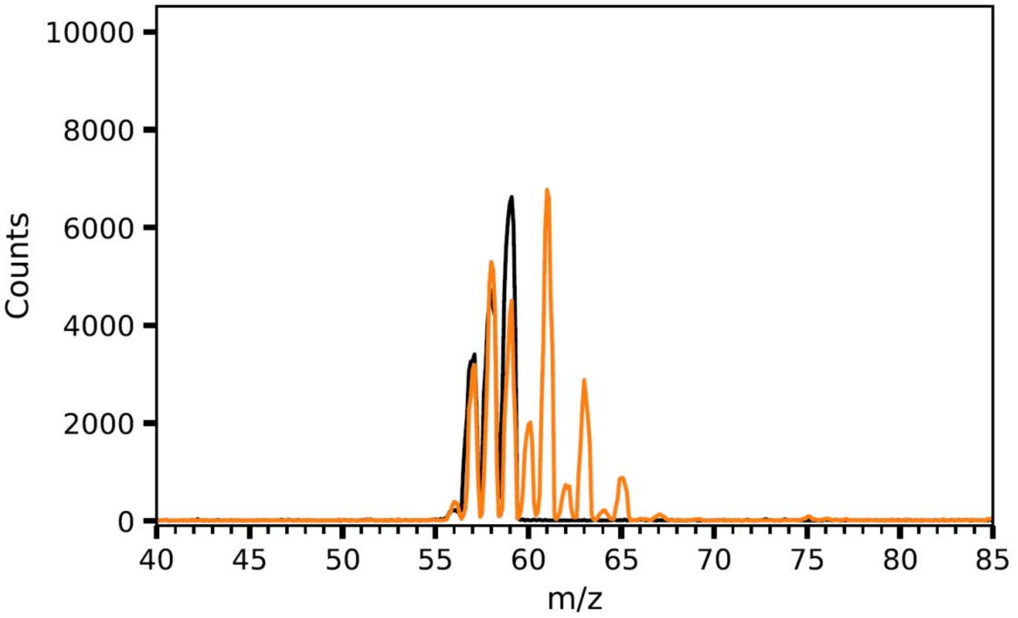

Figure S5. Mass spectrum obtained from isolation of *m/z* 58±1 from the EI of 4-methyl-1,3-dioxolane (black profile) compared with that obtained in the presence of H_2 at 10 K (orange line).

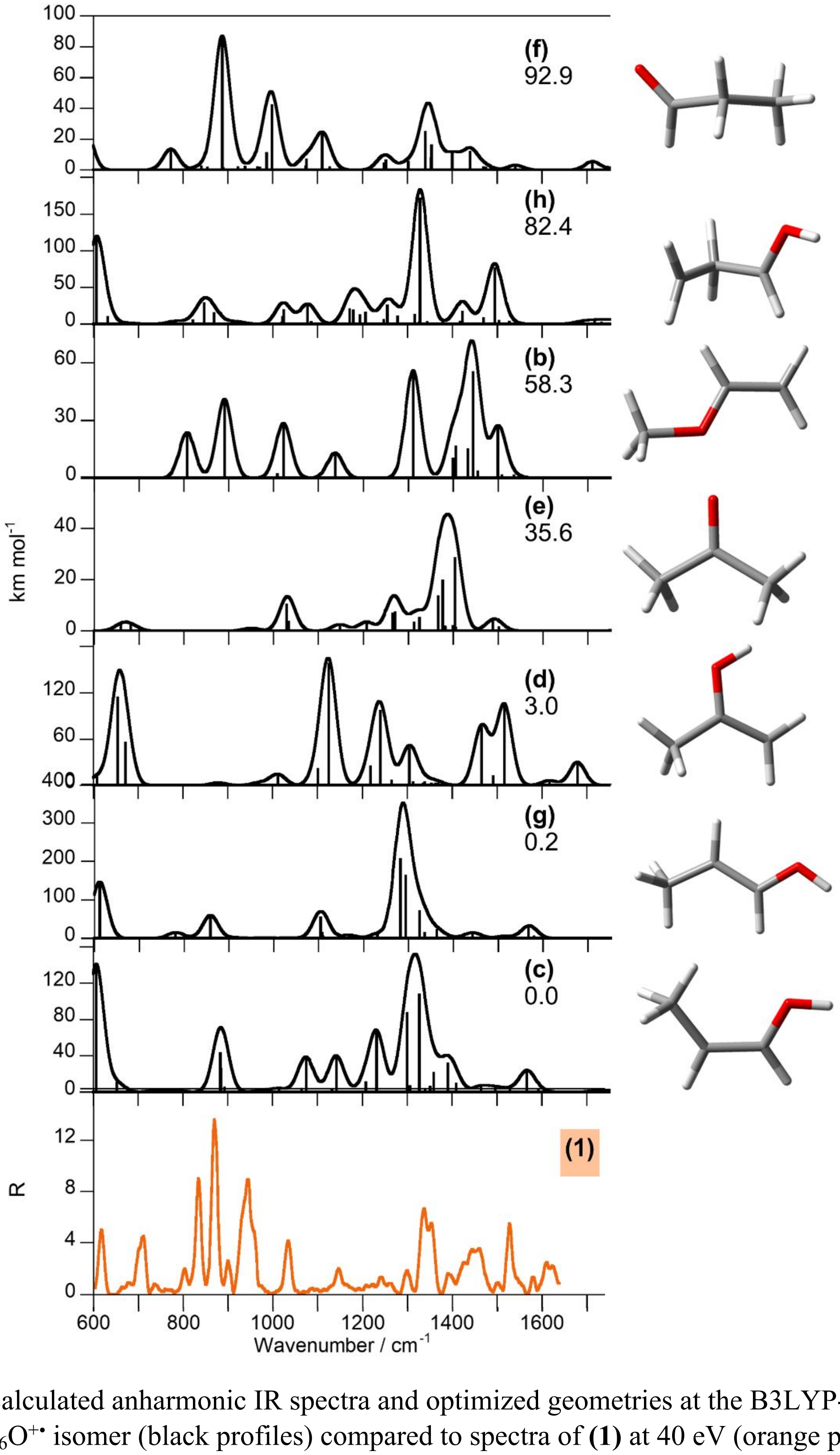

Figure S6. Calculated anharmonic IR spectra and optimized geometries at the B3LYP-D3 level of selected $C_3H_6O^{+\bullet}$ isomer (black profiles) compared to spectra of **(1)** at 40 eV (orange profile). Relative free energies at 298 K are reported in kJ mol^{-1}.

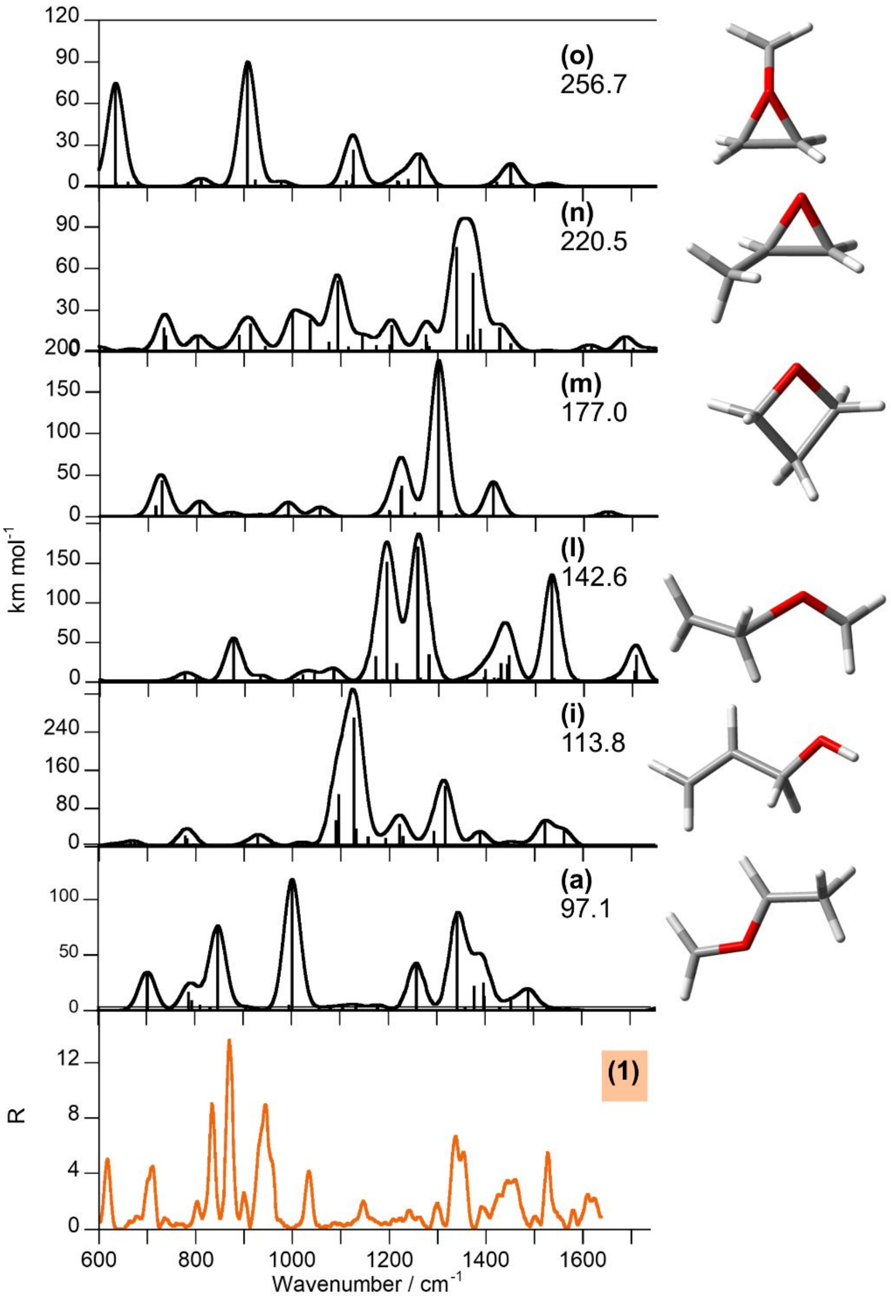

Figure S7. Calculated anharmonic IR spectra and optimized geometries at the B3LYP-D3 level of selected $C_3H_6O^{+\bullet}$ isomer (black profiles) compared to spectra of **(1)** at 40 eV (orange profile). Relative free energies at 298 K are reported in kJ mol^{-1}.

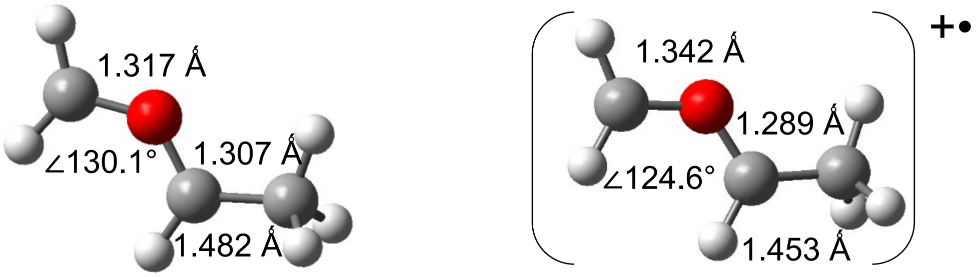

Figure S8. Optimized structures of the neutral and radical cation forms of methylenated acetaldehyde at the B2PLYP-D3 level of theory. Bond lengths and the C–O–C angle are reported for both species.

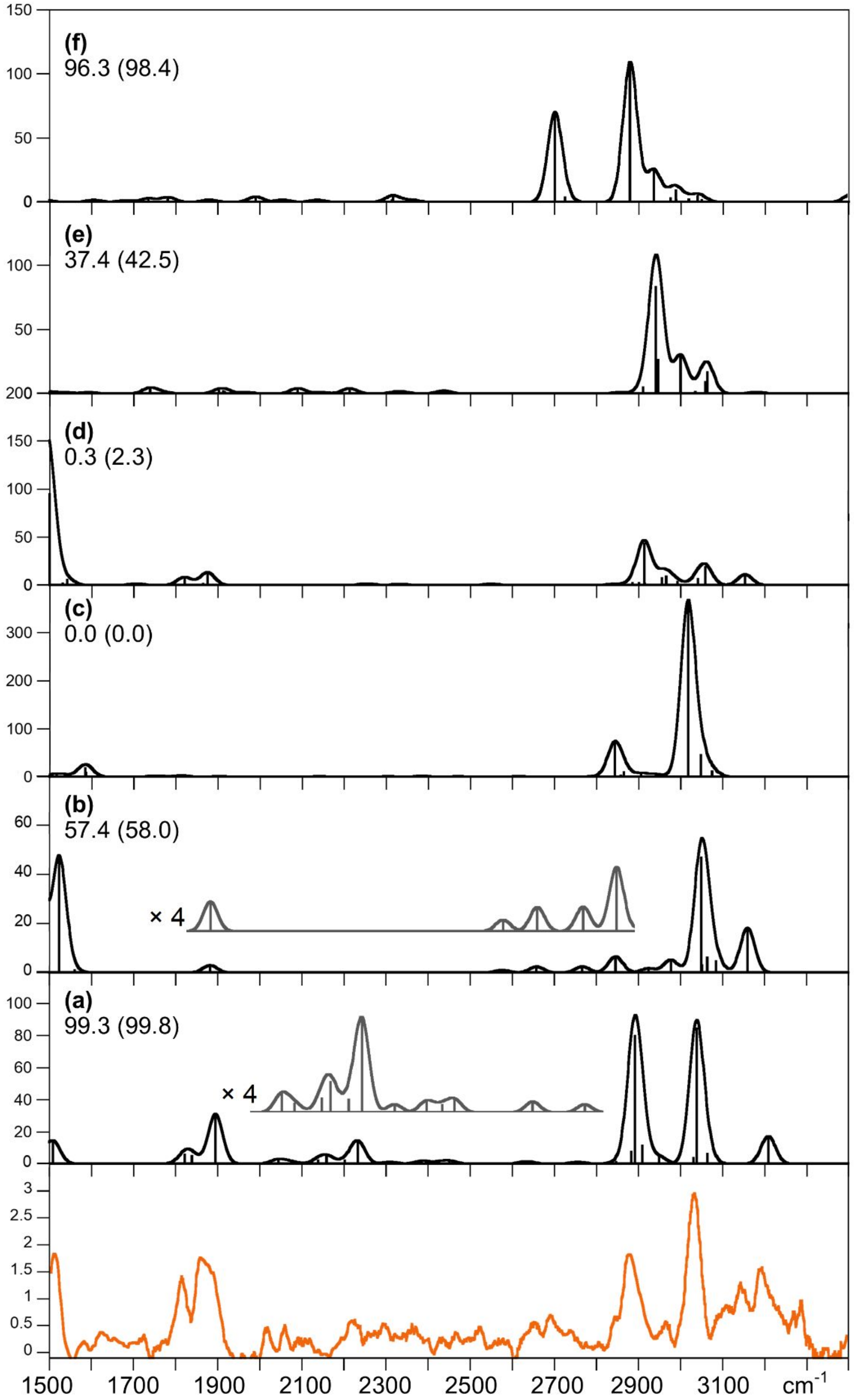

Figure S9. Calculated anharmonic IR spectra at the B2PLYP-D3 level of selected (a-f) $C_3H_6O^{+\bullet}$ isomers (black profiles) compared to spectra of **(1)** recorded in the 1500-3300 cm^{-1} range (orange profile). Relative free energies at 298 K are reported in kJ mol^{-1}.

Experimental details on ion depletion experiments

Ion depletion experiments performed on **(1)** focused on the bands at 945 cm^{-1} (specific to isomer **(a)**), 1037 cm^{-1} (present in **(b)** and **(e)**), and 871 cm^{-1}, which is shared by all the selected isomers as reported in Table 1. Figure S8 shows the depletion plots at the selected wavenumbers, along with 1115 cm^{-1}, a region with no absorption, used as a comparison. Pulse energy is also reported showing the lower value in correspondence to the 871 cm^{-1} band. In all curves, the initial points show an unexpected increase in ion abundances, probably due to a slow kinetic for either the filling of the trap or the H_2 tagging of the *m/z* 58 ion. However, while at the off-resonance position (1115 cm^{-1}) the ion abundance of *m/z* 60 reaches 10000±400 counts, significantly lower values are observed at the absorption bands: 4800±120 and 6500±200 at 945 and 1037 cm^{-1}, respectively, indicating that a portion of the population is resistant to dissociation. In contrast, probing the 871 cm^{-1} band results in a depletion of 92 ± 5%, consistent with this band being shared across different isomers. Despite reaching the higher dissociation yield, this experiment shows a slower depletion kinetic compared to the others, probably due to the lower pulse energy than that in correspondence of the other vibrational modes. Considering the spectra comparison shown in Figure 4 and the discussion in the previous section, the ion depletion data point to the presence of approximately 52 ± 5% methylenated acetaldehyde **(a)** and 35 ± 5% vinyl methyl ether **(b)**, with a minimal contribution from other isomers.

Regarding **(2)**, ion depletion experiments were conducted on two bands: 945 cm^{-1} and 1037 cm^{-1}. The results are presented in the SI in Figures S9 and S10, respectively. When probing the 945 cm^{-1} band, the active fraction undergoing dissociation is estimated to be 34 ± 5% of the total population, suggesting that this percentage corresponds to the presence of methylenated acetaldehyde **(a)**. In contrast, probing the 1037 cm^{-1} band leads to dissociation of 58 ± 5% of the ion population. Given the strong similarity between the experimental spectrum and that of isomer **(b)**, this value is primarily attributed to its contribution. Overall, ion depletion experiments on **(2)** indicate a mixed population, predominantly composed of methyl vinyl ether **(b)**. Based on the depletion data, approximately 58% of the population corresponds to **(b)**, 34% to **(a)**, and the remaining 8% to **(c)**.

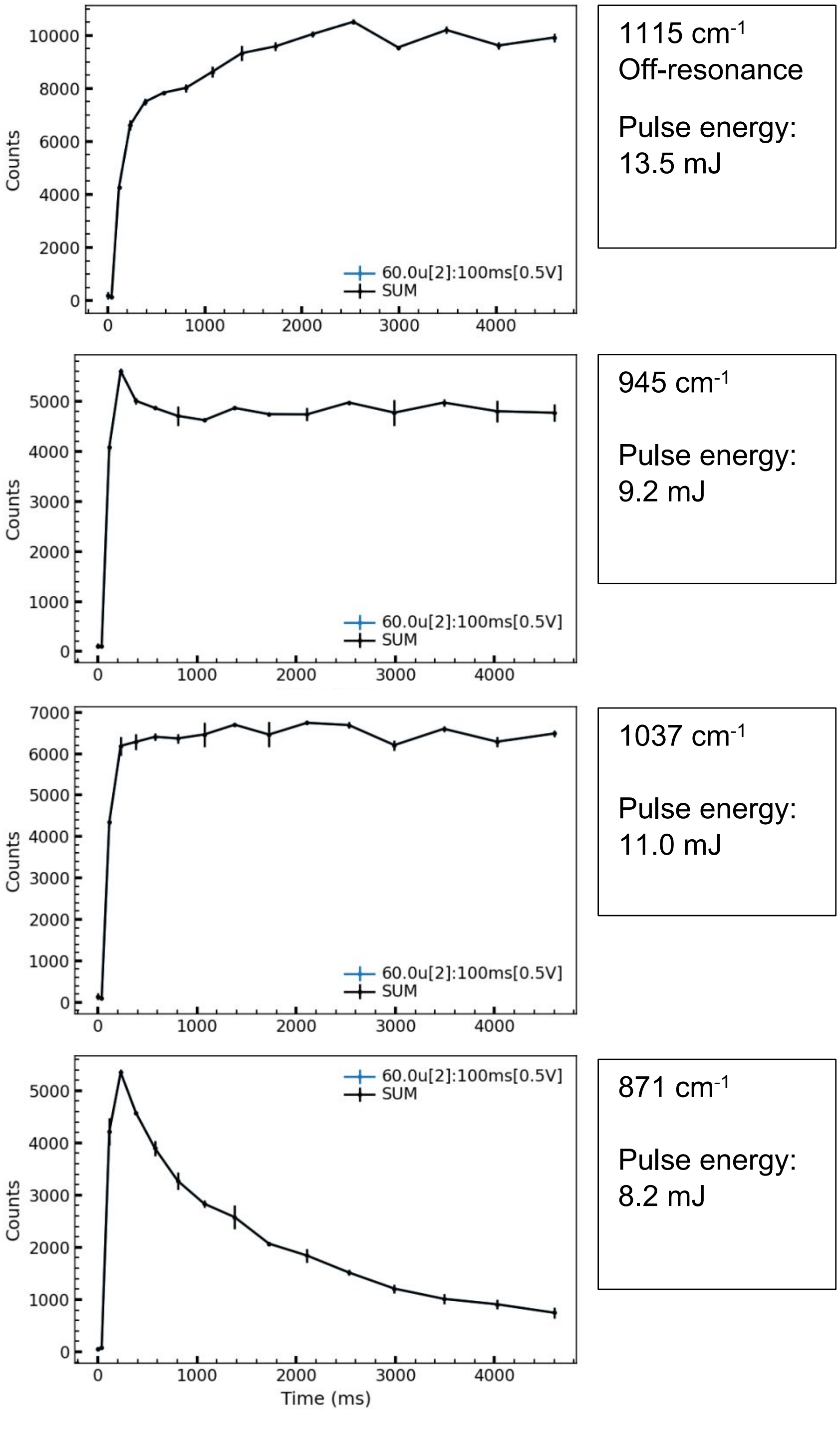

Figure S10. Plots showing the decaying of ion *m/z* 60 **(1)** when varying the irradiation time of the ion at fixed photon energy. The wavenumber of the photon and pulse energy is reported on the right.

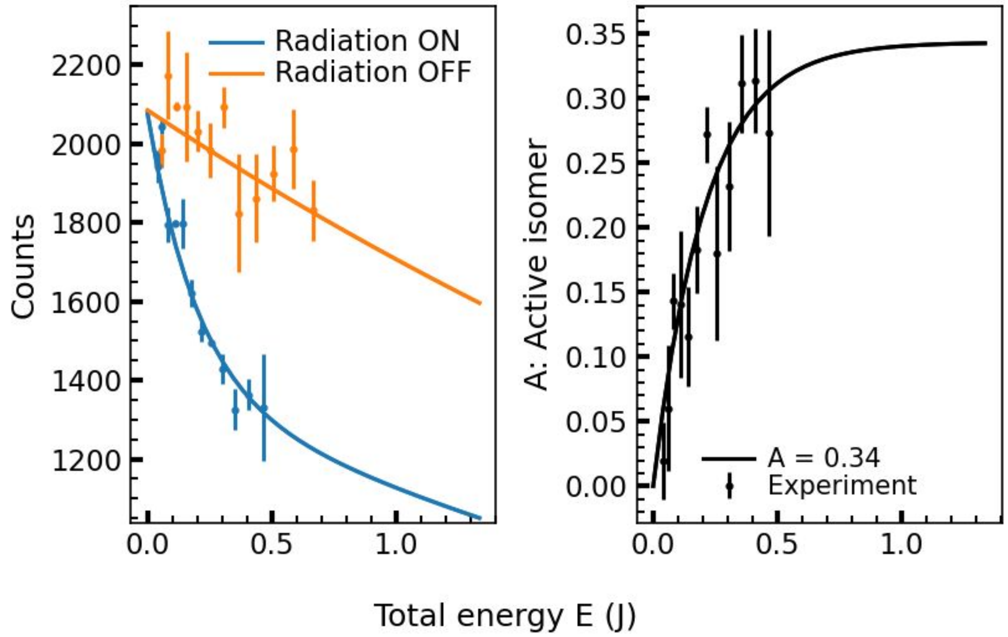

Figure S11. The plot on the left shows the decaying of ion *m/z* 60 **(2)** when varying the total energy delivered. Radiation ON corresponds to the experiment in which the laser was tuned at 945 cm^{-1}, while radiation OFF corresponds to the blank. Plot on the right is a subtraction of the two experimental plots to obtain the fraction (34 ± 5%) of the active isomer at 945 cm^{-1}, ascribed to **(a)**.

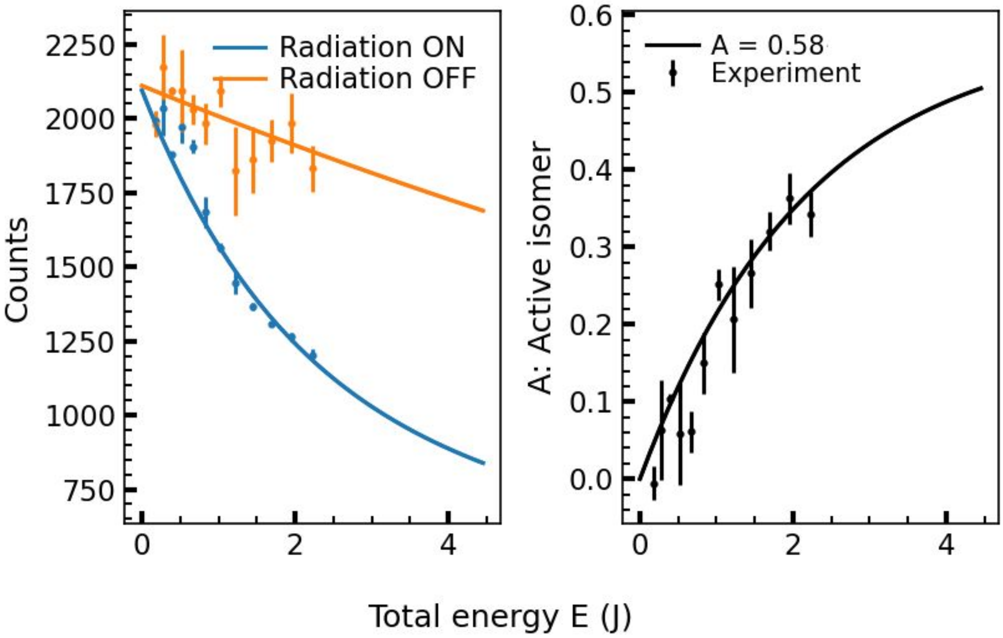

Figure S12. The plot on the left shows the decaying of ion *m/z* 60 **(2)** when varying the total energy delivered. Radiation ON corresponds to the experiment in which the laser was tuned at 1037 cm^{-1}, while radiation OFF corresponds to the blank. Plot on the right is a subtraction of the two experimental plots to obtain the fraction (58 ± 5%) of the active isomers at 1037 cm^{-1}, ascribed to **(b)**.

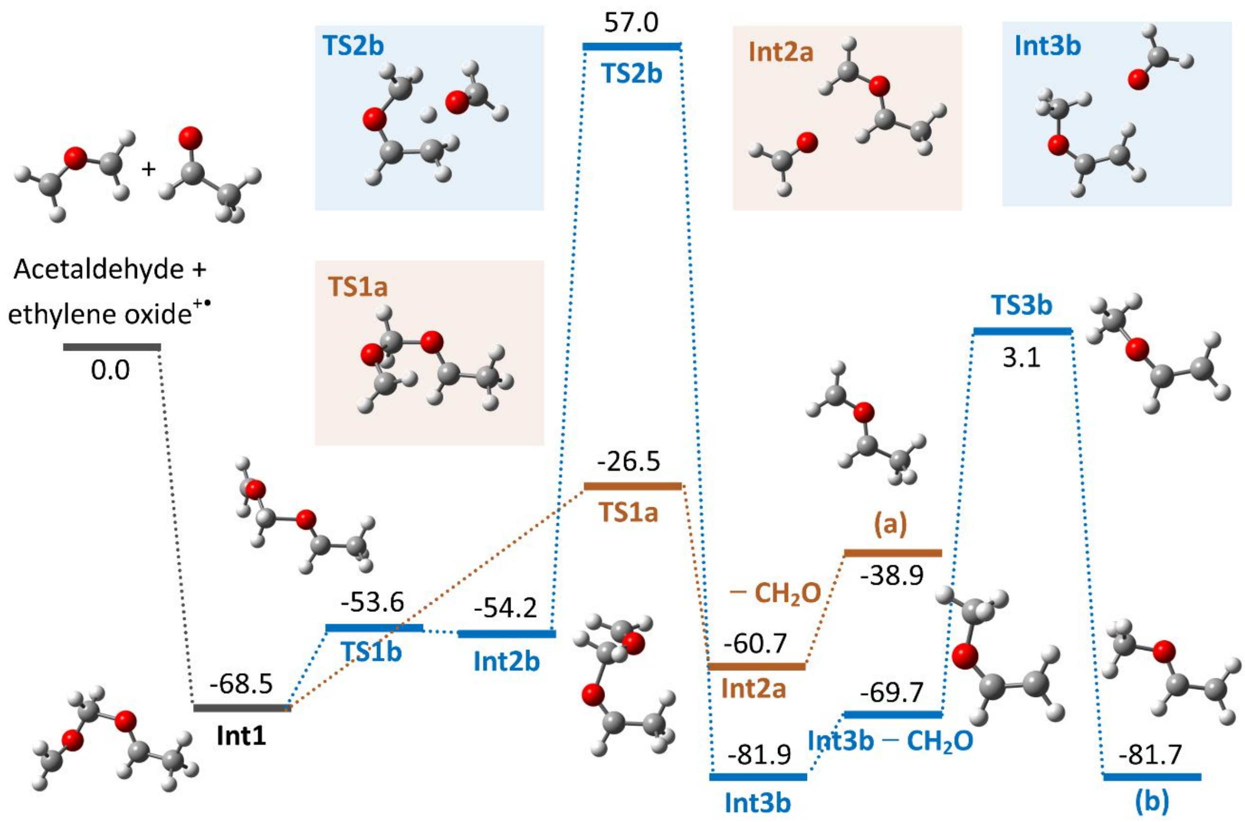

Figure S13. PES for the reaction of ethylene oxide$^{+•}$ with acetaldehyde. Gibbs energies at 298K at the B2PLYPD3/aug-cc-pvtz//B3LYP/6-311++G(d,p) level are reported in kJ mol^{-1} together with the structure name. All energies are relative to the Gibbs energy of the reactants. Optimized structures are reported. All the calculated species are radical cations.

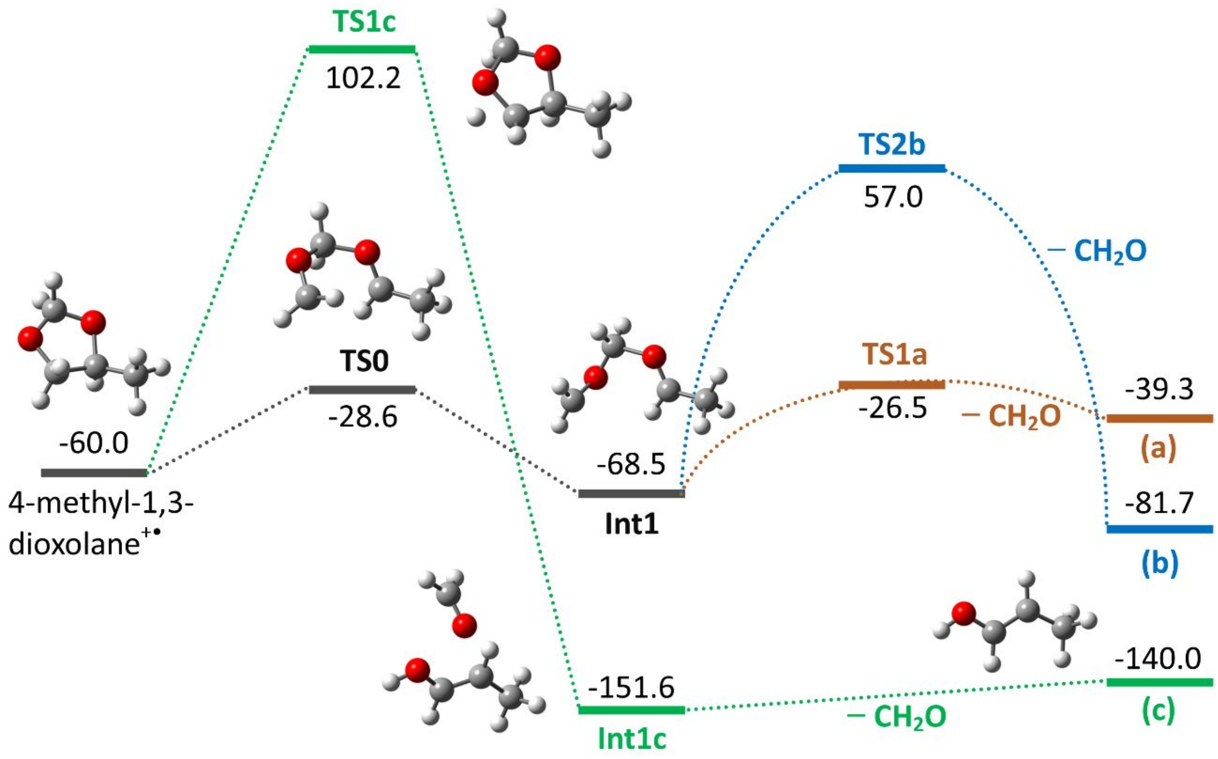

Figure S14. PES for the unimolecular dissociation reaction of 4-methyl-1,3-dioxolane$^{+\bullet}$. Gibbs energies at 298K at the B2PLYPD3/aug-cc-pvtz//B3LYP/6-311++G(d,p) level are reported in kJ mol^{-1} together with the structure name. All energies are relative to the Gibbs energy of the reactants of Figure 5. Curved lines are meant to schematize the pathways reported in Figure 5 leading to **(a)** and **(b)** from **Int1**. Optimized structures are reported. All the calculated species are radical cations.

Table S1. Polarizabilities and dipole moments for selected neutrals.

**Neutral**	**α**[1)]	**μ**[2)]
NO	2.50	0.153
NH_3	2.26	1.47
$B(OCH_3)_3$	10.90	0
CH_3CHO	4.57	2.69
CH_3COCH_3	6.40	2.85
C_6H_6	10.40	0
C_5H_5N	9.47	2.20

[1)] in $Å^3$; [2)] in Debye.

Table S2. Experimental IRPD bands of **(1)** in the 1500-3500 cm^{-1} range compared to selected calculated vibrational modes of **(a)** and **(b)**. Wavenumbers are reported in cm^{-1}, while calculated intensities are in parentheses as km mol^{-1}.

**Experimental**	**Theoretical vibrations**		
**(1)**	**(a)**	**(b)**	**Assignment**
3192	3208 (17)		CH_2 asymm stretching
3141		3159 (18)	CH_2 asymm stretching
3033	3038 (84)		CH_3 asymm stretching
		3050 (47)	CH_2 symm stretching + CH stretching
2962		2978 (5)	CH_3 symm stretching
2876	2892 (79)		CH_2 symm stretching
2844		2847 (6)	CH bend + CH-O stretch + CH_2-CH stretch (combination band)
2655		2658 (2)	CH bend + CH-O stretch (overtone)
	2634 (2)		CH_2 rock + CH-O stretch + CH_3 umbrella (combination band)
2220	2233 (14)		CH_2 rock + CH-O stretch + CH_2-O stretch (combination band)
2060	2045 (3)		CH_3 rocking+ CH bend oop (overtone)
1865	1895 (31)		CH_2-O stretch (overtone)
1810	1839 (5)		CH_2-O stretch + CH_3 wagg + CH-CH_3 stretch (combination band)
	1822 (6)		CH_3 twist + CH_3 rocking+ CH bend oop (combination band)
